# Histone demethylase KDM4C controls tumorigenesis of glioblastoma by epigenetically regulating p53 and c-Myc

**DOI:** 10.1038/s41419-020-03380-2

**Published:** 2021-01-18

**Authors:** Dong Hoon Lee, Go Woon Kim, Jung Yoo, Sang Wu Lee, Yu Hyun Jeon, So Yeon Kim, Hyeok Gu Kang, Da-Hyun Kim, Kyung-Hee Chun, Junjeong Choi, So Hee Kwon

**Affiliations:** 1 College of Pharmacy, Yonsei Institute of Pharmaceutical Sciences, Yonsei University, Incheon, 21983 Republic of Korea; 2 Department of Biochemistry and Molecular Biology, Yonsei University College of Medicine, Seoul, 03722 Republic of Korea; 3 Brain Korea 21 PLUS Project for Medical Science, Yonsei University College of Medicine, Seoul, 03722 Republic of Korea

**Keywords:** CNS cancer, Epigenetics, Targeted therapies

## Abstract

Glioblastoma is the most lethal brain tumor and its pathogenesis remains incompletely understood. KDM4C is a histone H3K9 demethylase that contributes to epigenetic regulation of both oncogene and tumor suppressor genes and is often overexpressed in human tumors, including glioblastoma. However, KDM4C’s roles in glioblastoma and the underlying molecular mechanisms remain unclear. Here, we show that KDM4C knockdown significantly represses proliferation and tumorigenesis of glioblastoma cells in vitro and in vivo that are rescued by overexpressing wild-type KDM4C but not a catalytic dead mutant. KDM4C protein expression is upregulated in glioblastoma, and its expression correlates with c-Myc expression. KDM4C also binds to the c-Myc promoter and induces c-Myc expression. Importantly, KDM4C suppresses the pro-apoptotic functions of p53 by demethylating p53K372me1, which is pivotal for the stability of chromatin-bound p53. Conversely, depletion or inhibition of KDM4C promotes p53 target gene expression and induces apoptosis in glioblastoma. KDM4C may serve as an oncogene through the dual functions of inactivation of p53 and activation of c-Myc in glioblastoma. Our study demonstrates KDM4C inhibition as a promising therapeutic strategy for targeting glioblastoma.

## Introduction

Glioblastoma is the most common primary brain cancer. Glioblastoma remains one of the most malignant diseases with limited treatment options and leads to lethality within two years of diagnosis^1^. Although genetic, environmental, bacterial infection, and several other factors are known to be involved in gliomagenesis, the underlying molecular mechanism remains poorly understood. Typically, glioblastoma has the following characteristics: rapid disease progression, high metastasis, poor prognosis, and high mortality rates^2^. In addition, it is difficult to improve the glioblastoma response to therapies targeting profoundly altered signaling pathways due to the intratumoral heterogeneity and the presence of cancer stem cells. Therefore, there exists an urgent and critical need to define the pathogenesis of glioblastoma in a better manner, to identify useful biomarkers, and to explore novel alternative therapeutic targets.

Mutation and abnormal expression of different histone demethylases (KDMs) have often been observed in human diseases, including cancers^3,4^, making them potential therapeutic targets. Although the roles of specific KDM in a given cancer type remain to be elucidated, the KDM4 family members are largely considered as oncogenes. KDM4A-D are histone H3 lysine 9 (H3K9) specific demethylases^5,6^. The oncogenic properties of the KDM4 family could arise from activating their targets by binding to their promoters and removing trimethyl (me3) and dimethyl (me2) groups from H3K9. KDM4A-C possesses a similar protein structure, including the jumonji domain (JmjN and JmjC), a tandem plant homeodomain (PHD), and a tandem Tudor domain, whereas KDM4D lacks the PHD and Tudor domains, which function as a histone reader domain^6^.

Although the role of KDM4C in glioblastoma is not well investigated, KDM4C has been reported to be an oncogene in other cancers. Among the KDM4s, the *KDM4C* gene, which is located on chromosome 9p24, is often amplified or overexpressed in various hematological and solid cancers, including lymphoma, breast cancer, prostate cancer, esophageal squamous cell carcinoma, sarcomatoid lung carcinoma, and medulloblastoma^5,7–15^. In normal and some cancer cells, KDM4C regulates cell proliferation by activating the target genes that are involved in cell signaling, cell cycle, and translation. Consistently, the depletion of KDM4C from cancer cells leads to the attenuation of cell growth^10,16–18^, whereas overexpression of KDM4C in human nontransformed mammary epithelial cells results in phenotypic changes of neoplastic transformation, including growth factor- and anchorage-independent proliferation^10^. The involvement of KDM4C in tumorigenesis has been further supported by demonstrating that KDM4C expression requires colonosphere formation in colon cancer cells through interaction with β-catenin^19^. Together, these studies indicate that KDM4C promotes tumorigenesis. Therefore, this enzyme has recently become an attractive drug target for cancer therapeutics^20,21^. However, till date, no potent and specific small-molecule inhibitor of the KDM4 family, including KDM4C, has been developed. Moreover, the expression and function of KDM4C and its mechanisms of action in glioblastoma are largely unknown.

In the present, we investigated the function of KDM4C in glioblastoma development and progression. We identified KDM4C as an essential factor required for the proliferation of glioblastoma and demonstrated that c-Myc and p53 are critical downstream effectors of KDM4C in this process. KDM4C positively correlated with c-Myc expression in human glioblastoma cell lines and tissues. KDM4C directly induced the expression of *c-Myc*. In addition, we revealed that KDM4C is a novel lysine demethylase for a tumor suppressor p53. KDM4C interacted with and demethylated p53 monomethylation at lysine 372 (p53K372me1), resulting in decreased p53 stability. Thus, the pro-apoptotic function of p53 was compromised in the presence of KDM4C. Our findings reveal that KDM4C promotes glioblastoma survival by inactivating the c-Myc-mediated p53 pathway and confirms that KDM4C can be a promising target for the treatment of glioblastoma.

## Materials and methods

### Reagents

SD70 was purchased from Xcess Biosciences (San Diego, CA, USA).

### Cell lines and culture

Human glioblastoma cell lines [wild-type (wt) p53; U87 (HTB-14) and A172 (CRL-1620), and mutant (mt) p53; LN229 (CRL-2611), SNB19 (CRL-2219), and T98G (CRL-1690)] were purchased from American Type Culture Collection (ATCC, Manassas, VA, USA). U343 (wt p53; 300365) and U251 (mt p53; 300385) cells were purchased from CLS Cell Lines Service GmbH (Eppelheim, Germany). LN215 (mt p53) cells were a kind gift from Dr. Erwin G. Van Meir (Emory University, Atlanta, Georgia, USA) and D54 (wt p53) cells from Dr. Andres Gutiérrez (Bringham, AL, USA). Cells were cultured in Dulbecco’s modified Eagle’s medium (DMEM) (Gibco, Gaithersburg, MD, USA) containing 10% fetal bovine serum (FBS), 100 units/ml penicillin, and 100 µg/ml streptomycin in a humidified atmosphere of 5% CO_2_ and 95% air at 37 °C. ATCC and CLS uses karyotyping, morphology, and PCR-based approaches to confirm the identity of cell lines.

### Lentiviral shRNA knockdown

The lentiviral shRNA sets for KDM4C were purchased from Sigma-Aldrich (St. Louis, MO, USA) following the procedure of Addgene (Watertown, MA, USA). The following sequences within human KDM4C were targeted: CGGGCAGAGAGTAATGGTGTGTTACTCGAGTAACACACCATTACTCTCTGCTTTTT (#1, NM_015061.1-1702s1c1, TRCN0000022058), CCGGGCCCAAGTCTTGGTATGCTATCTCGAGATAGCATACCAAGACTTGGGCTTTTT (#2, NM_015061.1-768s1c1, TRCN0000022054), and CCGGCAGCGGGTAGGGCGATAATTTCTCGAGAAATTATCGCCCTACCCGCTGTTTTTG (#3, NM_015061.2-4140s21c1, TRCN0000257272). The non-targeted control sequence (shCtrl NT) is GCAAGCTGACCCTGAAGTTCATTCAAGAGATGAACTTCAGGGTCAGCTTGCTTTTTG. To generate respective lentivirus, shRNA vector (pLKO.1) and packaging plasmid (psPAX2) and enveloping plasmid (pMD2.G) were used to cotransfect HEK293T cells as previously described^22^. Supernatants containing lentivirus were collected 48 and 72 h after transfection. Then, cells were infected three times every 12 h with lentivirus. Subsequently, cells were selected for 48 h in 2 μg/ml puromycin, to select the cells carrying the respective antibiotic resistance gene.

### Establishment of U87 Tet-On doxycycline-inducible shKDM4C cells

The Tet-on 3G doxycycline-inducible expression system (#631354, Takara Bio USA) was used to induce the expression of shKDM4C inserted under the TRE3G promoter in U87 cells according to the manufacturer’s protocol. U87 cells were treated with lentivirus-Tet3G (pLVX-EF1a-Tet3G regulator vector) and selected with G418 (2 mg/ml) to generate U87-Tet3G cells. Next, U87-Tet3G cells were stably transfected with pLVX-TRE3G-shKDM4C (shKDM4C sequences; #1, NM_015061.1-1702s1c1, TRCN0000022058 and #2, NM_015061.1-768s1c1, TRCN0000022054) response vector and selected with puromycin (2 μg/ml) for Tet-On inducible shKDM4C cells. The expression of KDM4C was confirmed in media containing doxycycline (Dox; 100 μg/ml).

### Small interfering RNA (siRNA) transfection

For siRNA transfection, we purchased control luciferase and KDM4C siRNA from ST Pharm Oligocenter (Seoul, Republic of Korea). The siRNA sequence targeted KDM4C: sense, 5′-GCAGCUGAGCAAGAGUAUA(dTdT)-3′ and anti-sense, 5′-UAUACUCUUGCUCAGCUGC(dTdT)-3′ and siRNA sequences targeted luciferase control: 5′-UCGAAGUACUCAGCGUAAG(dTdT)-3′. For transfection, cells were grown to 80% confluence and then transfected with siRNA (100 nM) RNAiMAX Lipofectamine agent (ThermoFisher, Waltham, MA, USA) according to the manufacturer’s instructions. Finally, cells were incubated with the siRNA mixture for 24 h and changed the medium with fresh serum-free medium.

### Cell growth and viability assay

Cell growth and viability were determined using the CCK8 assay [Cell Counting Kit (CCK)-8 kit, Dojindo Molecular Technologies, Inc., Kumamoto, Japan] as described previously^23^. Cells were seeded at a density of 0.5–1 × 10^3^ cells in 200 μl medium in 96-well plates in triplicate. After overnight incubation, SD70 was added to the cells at the indicated concentrations for 72 h at 37 °C. Twenty microliters of WST-8 was added to each well for the final 3 h, and absorbance was measured at 450 nm using a microplate reader (TECAN, Mannedorf, Switzerland). For cell growth analysis, a standard curve was generated using solutions of known cell counts (0.5 × 10^3^, 1 × 10^3^, 2 × 10^3^, 3 × 10^3^, 4 × 10^3^, 5 × 10^3^, 6 × 10^3^, 7 × 10^3^, and 8 × 10^3^ cells/well). Cell counts were indirectly estimated from absorbance measurements relative to the obtained standard curve. The absorbance of the negative control without DMSO was used to normalize data at each time interval. For cell viability analysis, the cell viable percentage was calculated relative to the negative control group. Results were statistically analyzed by Prism 5 (GraphPad Inc., San Diego, CA, USA). IC_50_ and GI_50_ values were calculated by nonlinear regression model.

### Proliferation assay

The effect of KDM4C knockdown on U87 and U251 cell proliferation was monitored in real-time using the IncuCyte^TM^ Live-Cell Imaging System (Essen BioScience, Hertfordshire, United Kingdom) as described previously^22^. A total of 500 cells expressing KDM4C shRNA were seeded in 96-well plates at a minimum of six replicates. The next day, automated phase-contrast images were obtained using IncuCyteTM microscope. Individual images were processed using an embedded contrast-based confluence algorithm that converts the monolayer confluence for each image at each time point to percentage. Multiple images were collected for each well and obtained a statistical measurement of confluence.

### Liquid colony formation assay

In total, 500 cells were seeded into 6 well plates and cultured for 24 h. Cells were then treated with shKDM4C or various doses of SD70 in triplicate. Drugs were removed at 96-h post-treatment, and cells were cultured until they reached optimal densities. Colony formation was visualized by staining the plates with 0.05% crystal violet (Sigma) for >1 h according to the manufacturer’s instruction. Integrated density was determined using the NIH ImageJ software.

### Apoptosis assay

Apoptosis was analyzed using the Annexin V/propidium iodide (PI) double-staining method according to the manufacturer’s instructions (Annexin V-FITC Apoptosis Detection Kit; BD Biosciences, Franklin Lakes, NJ, USA) as previously described^24^. After treatments, the cells were stained for 30 min at room temperature with 0.5 mg/ml Annexin V in binding buffer (10 mM HEPES free acid, 0.14 M NaCl, and 2.5 mM CaCl_2_). Next, 5 mg/ml of PI was added and incubated for 15 min at room temperature. The cells were assessed using a flow cytometer and BD FACSDiva software version 7 (both from BD Biosciences).

### Western blot analysis and co-immunoprecipitation (Co-IP) assay

As indicated, cells were grown, treated, collected, lysed, and separated by SDS-PAGE; western blotting was also performed as described previously^25^. Immunoprecipitation was achieved by incubating the cell lysates with anti-p53 monoclonal antibody (sc-126, Santa Cruz Biotechnology, Dallas, TX, USA) at 4 °C overnight with rotation. Protein A/G agarose slurry was added to the reaction mixture for 3 h. Then, the precipitated proteins were detected by immunoblotting with the corresponding antibodies. The blots were semi-quantified using the FusionCapt software version 16.08a (Vilber Lourmat Sté, Collégien, France). Protein expression levels were quantified against α-tubulin or GAPDH, and control levels were set at 1. The sources of the primary antibodies are listed in Supplementary Table 1.

### Luciferase reporter assay

U87 cells were seeded at 2 × 10^4^ into 24-well plates and allowed to reach ~70% confluence. Cells were transiently transfected with 300 ng of luciferase reporter constructs driven by the human p21 or Bax promoter using iN-fect (iNtRON Biotechnology, Seongnam, Republic of Korea). In addition, 50 ng of empty pcDNA3.1 or pcDNA3-p53 vectors and 300 ng of empty pCMV-HA or pCMV-HA-KDM4C vectors were cotransfected into U87 cells. After 24 h of transfection, the cell lysates were analyzed with a Luciferase Reporter Assay kit (Promega, Madison, Wisconsin, USA), according to the manufacturer’s instructions as previously described^23^. In all experiments, luciferase activity was analyzed at equal amounts of protein. Relative luciferase activity was measured by a microplate reader (TECAN, Maennedorf, Switzerland). All experiments were performed at least three times. The DNA constructs are listed in Supplementary Table 2.

### RNA isolation and quantitative reverse transcriptase-PCR (qRT-PCR)

RNA isolation, cDNA synthesis, and quantitative RT-PCR were performed according to the manufacturer’s instructions (Invitrogen, Carlsbad, CA, USA) as previously described^23^. qRT-PCR was performed with the Applied Biosystems 7500 System (Applied Biosystems, Foster City, CA, USA) using SYBR green qPCR master mix and gene-specific primers. mRNA expression levels were normalized to GAPDH mRNA levels. Fold expression determination, gene-to-GAPDH ratios were determined by using the 2^−ΔΔCt^ method. The primer sequences are listed in Supplementary Table 3.

### Chromatin immunoprecipitation (ChIP) assay

U87 cells were cross-linked using 1% formaldehyde for 10 min at room temperature. Nuclear extracts were prepared, and chromatin was sonicated to generate 200–1000 bp DNA fragments. For each assay, 40 μg DNA was immunoprecipitated by control IgG or KDM4C (ab13440, abcam), p53 (sc-126, Santa Cruz Biotechnology), histone H3K9me3 (ab8898, abcam), and histone H3K4me3 (07-473, Millipore) antibodies with protein A beads. The DNA-protein crosslinks were reversed by incubating at 65 °C for 4 h. Then, DNA was extracted using phenol and chloroform, followed by ethanol precipitation. Recovered and purified chromatin fragments were subjected to qPCR. IgG control experiments were performed for all ChIPs and incorporated into the IP/Input (10%) by presenting the results as (IP-IgG)/(Input-IgG). Corresponding primers: c-Myc P0 promoter, forward 5′-CCCCCGAATTGTTTTCTCTT-3′ and reverse 5′-TCTCATCCTTGGTCCCTCAC-3′, c-Myc P1 promoter, forward 5′-GAGGGATCGCGCTGAGTA-3′ and reverse 5′-CGGCTCTTCCACCCTAGC-3′, c-Myc P2 promoter, forward 5′-GCCGCATCCACGAAACTT-3′ and reverse 5′-TCCCCAAATGGGCAGAAT-3′, c-Myc P3 promoter, forward 5′-CGGTGCAGCCGTATTTCTAC-3′ and reverse 5′-CAGCAGCTCGAATTTCTTCC-3′, PUMA p53 binding promoter, forward 5′-ACAGCGCCTGGGTCCTCCTT-3′ and reverse 5′-GGACAAGTCAGGACTTGCAG-3′.

### Animal experiments

All animal experiments were approved by the Institutional Review Board of the Yonsei University College of Medicine and were performed in specific pathogen-free facilities according to the university’s guidelines for the Care and Use of Laboratory Animals. Mice were inoculated subcutaneously with 2 × 10^6^ stable TetOn-non-target (NT), TetOn-shKDM4C-1, and TetOn-shKDM4C-2 U87 cells into each flank under 100 µL of saline/zoletil/rompun (7:1:1) anesthesia. Randomized mice (*n* = 6 per group) were treated with doxycycline (Duchefa, Netherlands) after 13 days of tumor implantation. Doxycycline was administered to tumor-bearing animals orally, three times a week for 2 weeks at a dose of 10 mg/kg. Tumor sizes were measured every 3 to 4 days from palpable tumor formation until termination using calipers. Tumor volume was calculated using the following formula: length × width 2 × 0.5236. Mice were sacrificed in a 7.5% CO_2_ chamber, and the tumors were harvested for immunohistochemistry and other experiments.

### Immunohistochemistry (IHC) assay

For IHC assays, paraffin-embedded tissue sections were incubated with KDM4C (1:100, ab13440, abcam) and c-Myc antibodies (1:100, sc-40, Santa Cruz Biotechnology) at 4 °C overnight. SignalStain Boost IHC Detection Reagent (horseradish peroxidase, mouse) (Cell Signaling Technologies, Danvers, MA, USA) was added at room temperature for 10–30 min, according to the manufacturer’s instructions as previously described^23^. For visualization, DAB Horseradish Peroxidase Color Development Kit was used. For tissue counterstain, hematoxylin was used.

### Statistical analysis

Statistical analyses were performed with GraphPad Prism software (version 5.0, GraphPad Software). All data are presented as the mean ± standard deviation (SD) of more than three independent experiments. Statistical differences were determined by Student’s *t*-test, one-way analysis of variance (ANOVA) with post hoc analysis using Tukey’s multiple comparison test, and Pearson correlation coefficient tests. *P* < 0.05 was considered to indicate a statistically significant difference.

## Results

### KDM4C is upregulated in glioblastoma

Elevated KDM4 expression has been reported in various cancers and indicates poor prognosis in several cancer cases^26^. However, the role of KDM4 in glioblastoma tumorigenesis has not yet been well defined. To determine the expression level of KDM4 proteins in glioblastoma, we conducted mRNA expression profiling on glioblastoma microarray data from Gene Expression Omnibus (GEO) dataset (GSE90886) (Fig. S1). Among the 29 KDMs, KDM4C expression was the only one significantly higher in glioblastoma tissues compared to control ones. We next analyzed the expression of KDM4 proteins in the following 9 different glioblastoma cell lines: A172, D54, U343, U87, LN215, LN229, U251, SNB19, and T98G. Immunoblot analysis revealed that KDM4C and KDM4D protein levels were highly expressed in most of the glioblastoma cell lines, whereas the protein levels of KDM4A and KDM4B were very low or not detected (Fig. S2). Our results demonstrated that KDM4C was highly expressed in glioblastoma cells and tissues.

### KDM4C is essential for glioblastoma proliferation

**Fig. 1:**
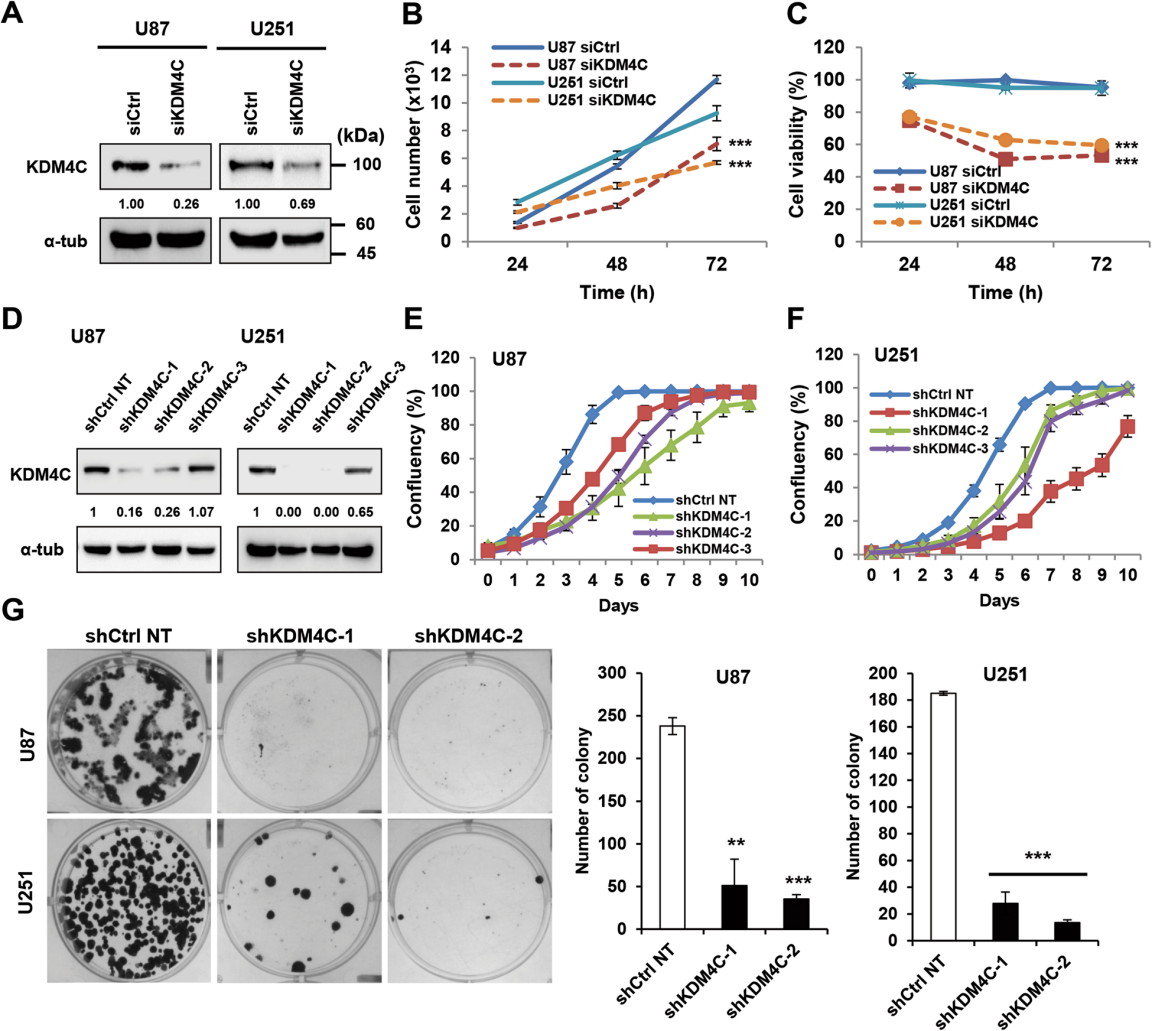
Selective inhibition of KDM4C suppresses glioblastoma cell growth and viability. **A**, **D**, **H** Immunoblotting of KDM4C expression in U87 and U251 cells treated. Normalized cell growth (**B**) and viability (**C**) of siCtrl and siKDM4C-treated U87 cells for 72 h. The proliferation of U87 (**E**) and U251 (**F**) cells expressing a non-target control shRNA (shCtrl NT) or shRNA constructs targeting KDM4C (shKDM4C) was monitored for 10 days using an IncuCyte ZOOM system. **G** Microscopic images and quantified colonies formed by KDM4C knockdown in U87 and U251 cells. Data present the mean ± SD (*n* = 3). *p < 0.05, **p < 0.01, and ***p < 0.001 vs. siCtrl or shCtrl NT control; Student’s *t*-tests.

**Fig. 2:**
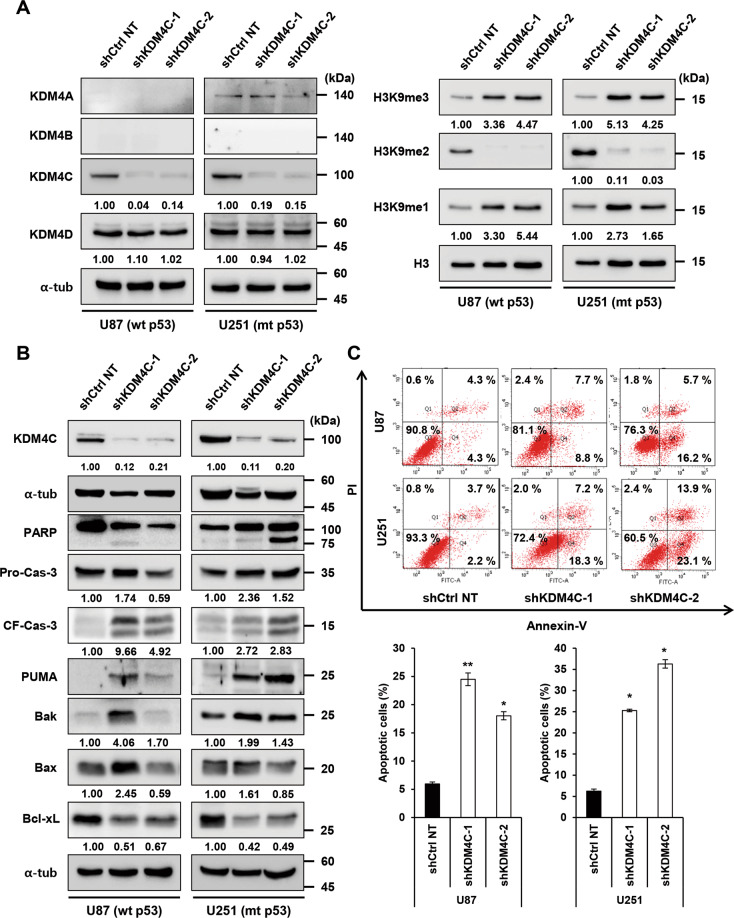
KDM4C knockdown triggers apoptosis in glioblastoma cells. **A** Immunoblotting of KDM4 and histone proteins in shCtrl NT and shKDM4C expressing U87 and U251 cells. **B** Immunoblotting of apoptotic markers in shCtrl NT and shKDM4C expressing U87 and U251 cells. **C** Apoptosis analysis of shCtrl NT and shKDM4C expressing U87 and U251 cells as determined by Annexin V/PI double-staining and flow cytometry. Data showing the percentage of early apoptotic and late apoptotic cells counted from dot plots. Data present the mean ± SD (*n* = 3). *p < 0.05, **p < 0.01, and ***p < 0.001 vs. shCtrl NT control; Student’s *t*-tests.

Based on the overexpression of KDM4C in glioblastoma, we speculated that KDM4C might regulate the proliferation of glioblastoma cells. To address the importance of KDM4C in glioblastoma cell growth and viability, we downregulated KDM4C in U87 and U251 glioblastoma cells using siRNAs or shRNA (Fig. 1A, D and Fig. S3A, D). Next, the proliferation kinetics of glioblastoma cells were assessed using IncuCyte and CCK8 assay. The growth curve revealed that knockdown of KDM4C in glioblastoma cells resulted in significant short-term (Fig. 1B and Fig. S3B, E) and long-term (Fig. 1E, F) growth inhibition compared to that in the control groups of the two cell lines. In addition, the CCK8 assay confirmed that the downregulation of KDM4C induced a significant decrease in cell viability (Fig. 1C and Fig. S3C, F). Similar results were also observed in other glioblastoma A172 cells (Fig. S3G‒I). Furthermore, we detected a pronounced inhibitory effect of KDM4C silencing and inhibition by SD70, a KDM4C-selective inhibitor^27^ on cell proliferation through the colony formation assay (Fig. 1G and Fig. S5). Notably, KDM4C knockdown resulted in increased global levels of H3K9me3 and H3K9me1, whereas there was a decline in the global levels of H3K9me2 (Fig. 2A). The above data were also confirmed by the treatment of SD70 in the glioblastoma cells (Fig. S4A), wherein KDM4C inhibition decreased the growth and viability of various glioblastoma cells both time- and dose-dependently (Fig. S4C, D and Fig. S6). Furthermore, the inhibitory effect of shKDM4C on the cell growth and viability was rescued by wt KDM4C but not by demethylase dead mutant KDM4C H190A, suggesting that the activity of KDM4C is essential for glioblastoma cell proliferation and survival (Fig. S7B, C).

### Depletion of KDM4C induces apoptosis in glioblastoma

To gain molecular insights into the mechanism by which KDM4C regulates glioblastoma growth and viability, we investigated the expression of key cell cycle and apoptotic regulatory proteins using immunoblot assays. Interestingly, we observed that KDM4C knockdown reduced expression of the cyclins (cyclin A1, D1, D2, and E2) and cyclin-dependent kinases (CDKs) (CDK2, CDK4, and CDK6) and induced the CDK inhibitor p21 (Fig. S8). In addition, knockdown of KDM4C induced the apoptosis of glioblastoma cells, as demonstrated by the increase in annexin V/PI-positive cells and upregulation of cleaved caspase-3 and cleaved poly(ADP ribose) polymerase (PARP) (Fig. 2B, C, and Fig. S9). Consistently, knockdown and inhibition of KDM4C increased the expression of proapoptotic markers (Bak, Bax, and Puma) and decreased the expression of an antiapoptotic marker (Bcl-xL) (Fig. 2B and Fig. S4B). We conclude that KDM4C depletion promotes apoptosis in glioblastoma cells.

### KDM4C oncoprotein upregulates c-Myc gene expression by directly binding to its promoter

**Fig. 3:**
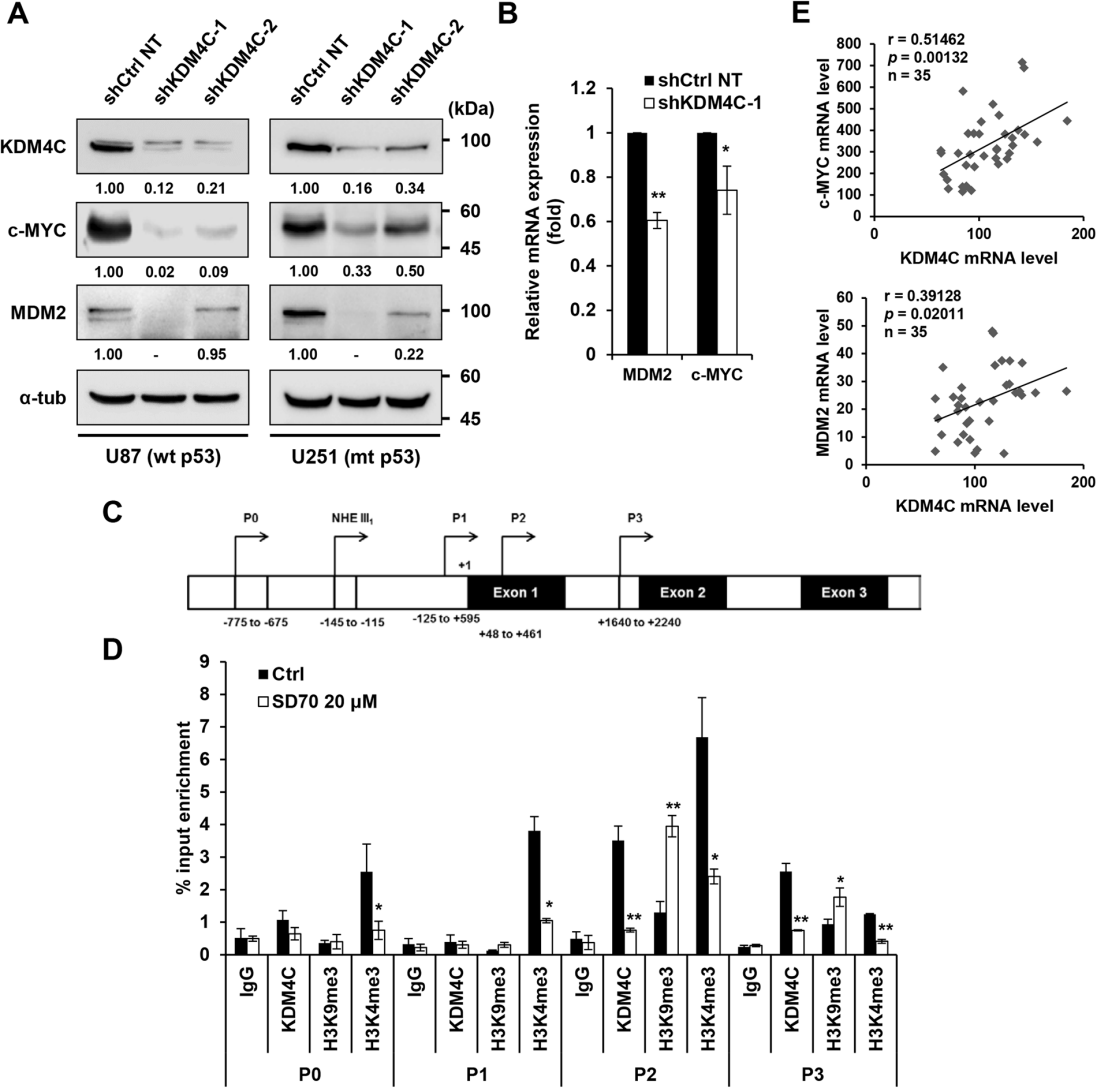
KDM4C depletion suppresses the expression of c-Myc in glioblastoma cells. **A** Immunoblotting of KDM4C and its target genes in shCtrl NT and shKDM4C expressing U87 and U251 cells. **B** RT-qPCR showing KDM4C and MDM2 mRNA levels in shCtrl NT and shKDM4C expressing U87 cells. mRNA expression levels were normalized to GAPDH mRNA levels. *p < 0.05 and **p < 0.01 vs. shCtrl NT control; Student’s *t-*tests. **C** Schematic representation of the c-Myc promoter. **D** ChIP-qPCR analysis of KDM4C, H3K4me3, and H3K9me3 levels at the c-Myc promoter in 0.1% DMSO (control)- and SD70-treated U87 cells. Data present the mean ± SD (*n* = 3). *p < 0.05 and **p < 0.01 vs. DMSO control; Student’s *t-*tests. E GEO analysis of microarray dataset GSE36245 (GPL570, glioblastoma brain tumors, *n* = 35). Pearson’s correlation coefficient a*n*alysis was used to measure the relationship between KDM4C and c-Myc or MDM2 mRNA levels.

c-Myc accumulates in both U87 glioblastoma cell line^28^ and tissues^29^. Before our study, Myc^18^ and MDM2^30^ were the few characterized target genes of KDM4C. KDM4C is known to promote gene transcription by erasing H3K9me2 marks from the target gene promoter, which is often related to gene silencing. We examined whether KDM4C depletion affects the expression of its target genes in glioblastoma cells. Our results showed that the expression levels of the KDM4C target genes *c-Myc* and *MDM2* significantly decreased after KDM4C knockdown (Fig. 3A, B and Fig. S10A). Treatment of SD70 decreased *c-Myc*, but not *MDM2* (Fig. S10). In contrast, KDM4C overexpression induced the expression of c-Myc in glioblastoma cells (Fig. S7A).

**Fig. 4:**
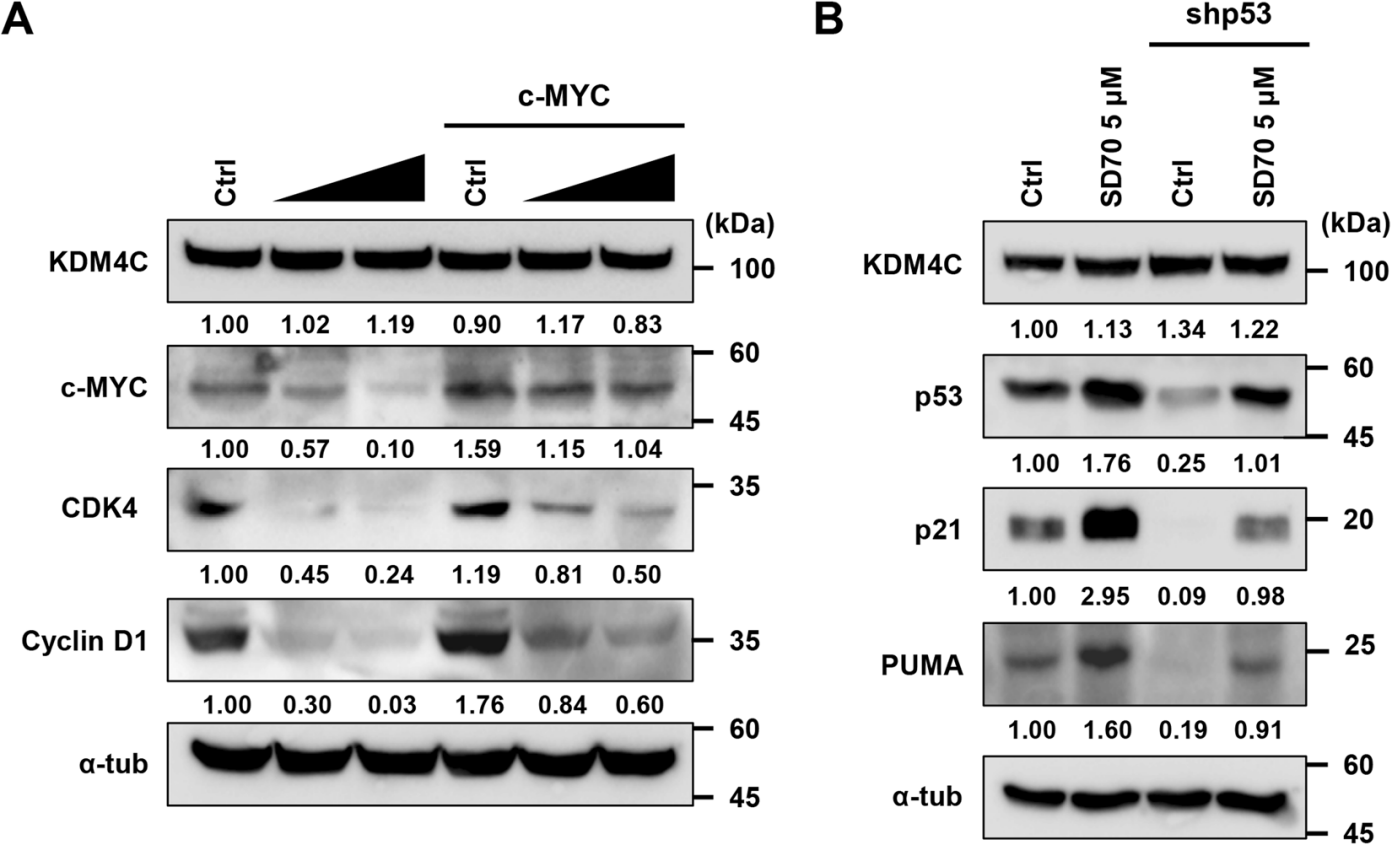
Altered expression of c-Myc and p53 in KDM4C-inhibited cells rescues c-Myc and p53 target gene expressions caused by KDM4C inhibition. **A** Immunoblot analysis of KDM4C, c-Myc, and its target genes in U87 cells transfected with c-Myc (0.6 μg) without or with the treatment of SD70 (10, 20 μM). **B** Immunoblot analysis of KDM4C, p53, and its target genes in shCtrl NT and shp53 expressing U87 cells without or with the treatment of SD70 (5 μM).

Next, to gain mechanistic insight into how KDM4C regulates the expression of its target gene, we performed ChIP assays in U87 cells to examine whether KDM4C is bound to the promoter regions of the *c-Myc* gene. The ChIP assays showed that KDM4C was significantly enriched on the *c-Myc* P2 and P3 promoters (Fig. 3C, D). In contrast, KDM4C binding was reduced at the *c-Myc* promoter in SD70-treated cells compared to that in control cells (Fig. 3D). This resulted in more enriched H3K9me3 and less enriched H3K4me3 at the *c-Myc* promoter (Fig. 3D), leading to the reduced expression of c-Myc. c-Myc, in turn, downregulated its known target genes CDK2, CDK4, cyclin D1, and cyclin E2 required for cell cycle control (Fig. S8). Furthermore, overexpression of c-Myc in KDM4C-inhibited cells abolished the reduction of its target gene expression caused by KDM4C expression (Fig. 4A). In parallel, an analysis of GEO profiles (GSE36245) in 35 patients with glioblastoma revealed a positive correlation between the expression of *KDM4C* and *c-Myc* (Fig. 3E). No statistically significant correlation was observed between other *KDM4s* and *c-Myc* levels (Fig. S11A). Another KDM4C target gene, *MDM2*, exhibited a similar expression correlation pattern in glioblastoma samples (Fig. 3E). Altogether, our results suggest that KDM4C epigenetically promotes the expression of the oncogene *c-Myc* by directly binding to its promoter and erasing H3K9me3 marks in glioblastoma cells.

### KDM4C interacts with p53 via its middle domains

Studies have shown that p53 interacts with KDM4A and KDM4C in human HCT116 colorectal cancer cells^31,32^. We speculated that KDM4 might interact with p53 in glioblastoma cells. We observed that KDM4A and KDM4C, but not two other proteins KDM4B and KDM4D, formed complexes with p53 at the endogenous protein levels in U87, LN215, and SNB19 glioblastoma cell lines (Fig. S12A, B). Next, we carried out reverse order Co-IP experiments and confirmed that p53 forms a complex with KDM4C. Subsequently, we conducted GST pull-down assays to define the domains of KDM4C and p53 that mediate their interaction. Although N-terminal 350 amino acids, which include the catalytic domain of KDM4C, did not interact with p53, the middle portion of KDM4C (amino acids 280–720) interacted with p53 (Fig. S12C, E). In addition to the full-length p53, the middle portion of p53 embracing its DNA-binding domain bound to KDM4C in vitro (Fig. S12D, F). Altogether, these results demonstrate that KDM4C directly interacts with p53.

### KDM4C inhibition promotes p53 transcriptional activity

**Fig. 5:**
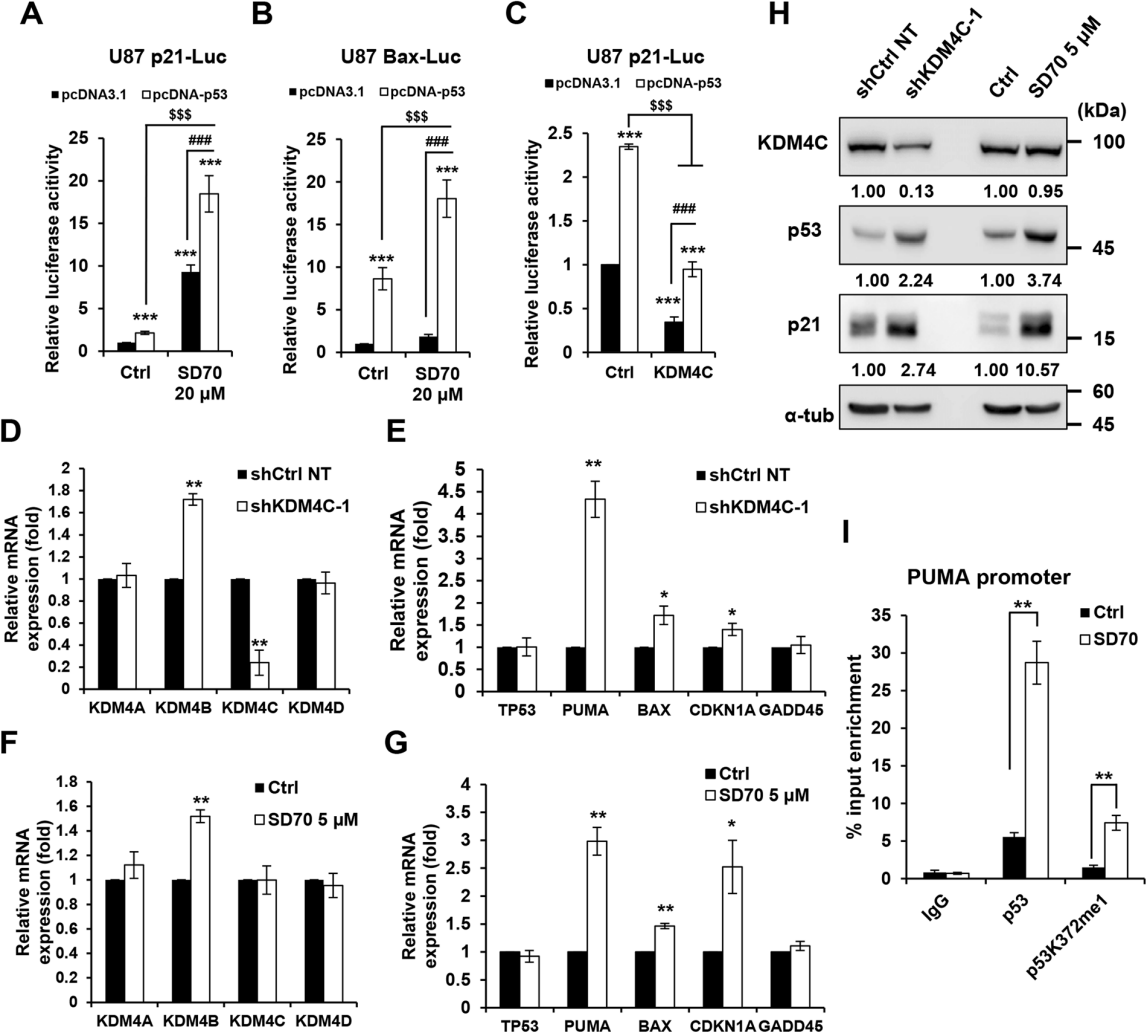
KDM4C inhibition promotes p53 transcriptional activity. **A**, **B** The activity of p21 and Bax luciferase reporter constructs in U87 cells treated with 0.1% DMSO (control) and SD70 following transfection with p53. ***p < 0.001 vs. pcDNA control; ^$$$^*p* < 0.001 vs. pcDNA-p53 group, ^###^*p* < 0.001 vs. DMSO^+^pcDNA-p53 group; one-way ANOVA. **C** The activity of a p21 luciferase reporter construct in U87 cells following transfection with p53 and KDM4C. **D**–**G** RT-qPCR showing KDM4s, p53, and p53 target gene mRNA levels in U87 cells treated with shRNA (**D** and **E**) or SD70 (**F** and **G**). mRNA expression levels were normalized to GAPDH mRNA levels. **H** Immunoblotting of KDM4C, p53, and p21 expression in U87 and U251 cells treated with shRNA or SD70 for 24 h. **I** ChIP-qPCR analysis of p53 and p53K372me1 levels at the PUMA promoter in 0.1% DMSO (control) and SD70 (5 µM) treated U87 cells. Data present the mean ± SD (*n* = 3). *p < 0.05 and **p < 0.01 vs. shCtrl NT or DMSO control; Student’s *t-*tests.

KDM4C forms complex with p53, which implies that KDM4C might inhibit p53 transcriptional activities by demethylating p53. Previous literature has demonstrated that lysine methylation of p53 occurs at K370, K372, K373, and K382 in the C-terminal regulatory domain^33^. p53 Methylation at K370, K373, and K382 suppresses p53 transcriptional activity, whereas methylation at K372 facilitates its activity. We investigated whether KDM4C activates or represses p53-dependent transcription using p21 or Bax promoter-luciferase assay in U87 cells. Our results showed that cotransfection of p53 promoted an increase in p21 and Bax promoter activities following KDM4C inhibition by SD70 treatment (Fig. 5A, B). This p53-mediated stimulation was blocked by the overexpression of KDM4C (Fig. 5C), suggesting that KDM4C inhibits p53 function. To further confirm this result, we examined whether direct genetic or pharmacological inhibition of KDM4C affects the expression of the p53 target genes. We observed that KDM4C knockdown and inhibition increased the expression of p53 target genes *CDKNA1* (p21), *PUMA, BAX*, and *KDM4B* in U87 cells (Fig. 5D–G). Consistently, the expressions of p21, Bax, and PUMA proteins were increased by KDM4C inhibition (Figs. 5H and 2C). In addition, the KDM4C inhibition in p53-depleted cells abrogated the induction of p53 apoptotic gene expression caused by KDM4C inhibition (Fig. 4B). Moreover, the ChIP assays revealed that KDM4C inhibition significantly increased the recruitment of p53 to the *PUMA* promoter in U87 cells compared to that in control cells (Fig. 5I). These data indicate that KDM4C regulates the transcriptional activity of p53.

### KDM4C demethylates p53 at lysine 372

**Fig. 6:**
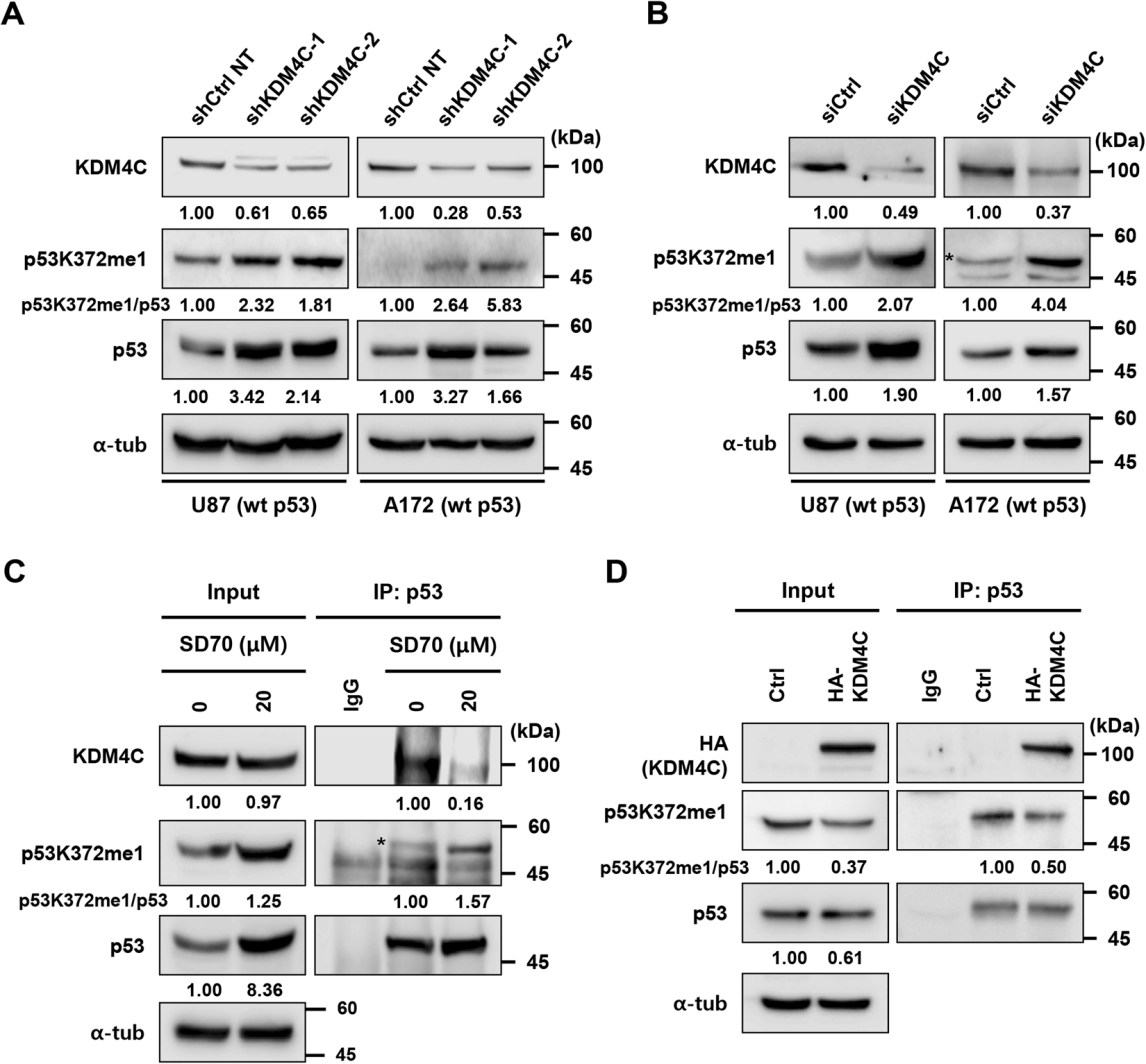
KDM4C interacts with and demethylates p53 at K372. **A**, **B** Immunoblotting of KDM4C, p53, and p53K372me1 in U87 and U251 cells transfected with (**A**) shRNA and (**B**) siRNA. **C**, **D** Lysates of cells treated with SD70 (20 μM) (**C**) or HA-KDM4C (**D**) were immunoprecipitated with p53 antibody followed by immunoblotting with antibodies as indicated. Ten percent of total cell lysates used in immunoprecipitation is shown as input. The level of p53K372me1 was quantified relative to p53, and the control levels were set at 1. The asterisk indicates the expected sizes for p53K372me1.

As KDM4C knockdown activated the expression of p53 target genes, we hypothesized that increasing the methylation of p53 enhances its transcriptional activities. Monomethylation of p53 at K372 (p53K372me1) by Set7/9 is required for the nuclear localization and stabilization of the chromatin-bound p53 for the transcriptional activation of target genes^33^. However, it remains largely unknown whether KDM4C regulates p53K372me1. Therefore, we determined whether KDM4C depletion increased the level of p53K372me1. We observed that KDM4C knockdown using shRNA and siRNA elevated the levels of p53K372me1 as well as p53 in wt p53 harboring U87 and A172 cells (Fig. 6A, B). Similarly, SD70 treatment also elevated the levels of p53K372me1 and p53 in wt p53 harboring U87 and A172 cells (Fig. S13). We also conducted IP to enrich p53K372me1 from whole-cell lysates and subsequently detected them using anti-p53 antibodies. As shown in Fig. 6, KDM4C inhibition by SD70 treatment dramatically increased the level of p53K372me1, whereas KDM4C overexpression produced the opposite effect in U87 cells (Fig. 6C, D). The increased p53K372me1 levels, in turn, promoted acetylation at the nearby C-terminal lysine residue of p53 (K381 and K382) and phosphorylation at serine 15, resulting in the stabilization of p53 protein. While acetylation levels of p53K381 and p53K382 increased, those of p53K372 decreased in both KDM4C-depleted and SD70-treated U87 cells (Figs. S13 and S14). To further validate whether the stabilization of chromatin-bound p53K372me1 increases the level of p53 on the *PUMA* promoter, we also conducted ChIP assays using anti-p53K372me1 antibodies. We observed that KDM4C inhibition indeed resulted in increased binding of p53K372me1 on the *PUMA* promoter (Fig. 5I). Collectively, these results suggest that KDM4C demethylates p53K372me1 to inhibit its transcriptional activity and proapoptotic functions.

### KDM4C is required for glioblastoma tumorigenesis

**Fig. 7:**
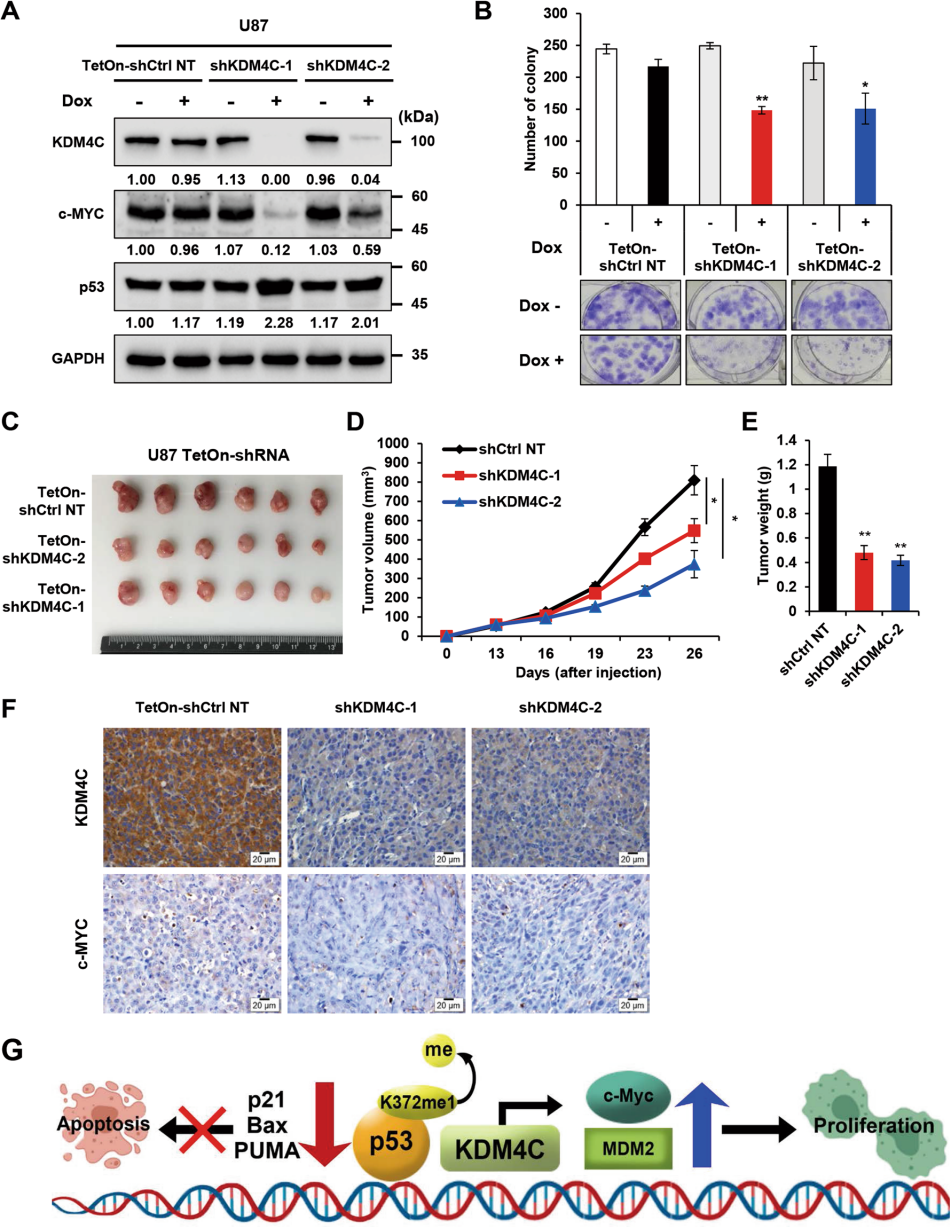
KDM4C regulates the proliferation and xenograft tumor growth of glioblastoma. **A** Immunoblotting of KDM4C, c-Myc, and p53 in U87 TetOn-KDM4C cells expressing shCtrl NT or shKDM4C. **B** Liquid colony formation assays of U87 TetOn-KDM4C cells expressing shCtrl NT or shKDM4C. *p < 0.05 and **p < 0.01 vs. Dox (-) group; one-way ANOVA. **C**–**E** Xenograft assays of U87 cells with inducible expression of shCtrl NT or shKDM4C (*n* = 3). **C** Representative U87 xenograft tumors isolated from individual animals. **D** Increase in tumor volume over time. **E** Final tumor weights of the U87 xenografts. Data present the mean ± S.D. *p < 0.05 and **p < 0.01 vs. shCtrl NT control; one-way ANOVA. **F** Representative image of IHC staining of KDM4C and c-Myc in tissues from U87 xenograft model. Scale bar, 100 μm. **G** Molecular model describing that KDM4C can regulate glioblastoma proliferation and apoptosis by modulating c-Myc and p53.

To investigate whether KDM4C is involved in the tumorigenesis of glioblastoma, we used a doxycycline-inducible (Tet-On) KDM4C shRNA system in U87 cells. Upon inducible KDM4C knockdown, the protein levels of KDM4C and c-Myc were significantly decreased, whereas the levels of p53 were increased in the doxycycline-treated groups compared to those in the control groups (Fig. 7A). Furthermore, the doxycycline-induced knockdown of KDM4C resulted in significant inhibition of cell growth and viability (Fig. S15). The clonogenic assay data revealed that the doxycycline-induced knockdown of KDM4C substantially reduced the colony formation of U87 cells (Fig. 7B). We next evaluated whether KDM4C knockdown affected tumor growth in vivo using the mouse tumor xenograft model. Our results showed that the tumorigenic capacity of U87 cells was hampered by the depletion of KDM4C (Fig. 7C–E). Quantification of tumor volume and tumor weight disclosed a significant reduction in tumor growth in mice xenografted with doxycycline-inducible shKDM4C U87 cells (Fig. 7C–E). These data indicate that KDM4C is essential for the tumorigenesis of glioblastoma.

## Discussion

Glioblastoma is the deadliest clinical subtype of glioma and one of the most poorly characterized glioma at the molecular level^1^. The poor understanding of this malignancy has significantly limited its therapeutic management. Although epigenetic dysregulation by KDM4C has emerged as an important mechanism in several cancers, the molecular mechanisms underlying the contribution of KDM4C to cell proliferation and tumorigenesis remain to be further elucidated in glioblastoma. In this study, we observed that KDM4C promotes the proliferation and tumorigenesis of glioblastoma through the activation of c-Myc and the inactivation of p53 (Fig. 7G). These findings demonstrate a novel role for KDM4C in controlling glioblastoma proliferation and tumorigenesis and suggest the potential therapeutic significance of KDM4C in glioblastoma.

Our findings demonstrate that the histone demethylase KDM4C is essential for the tumor progression of glioblastoma. To regulate this process, KDM4C engages two distinct mechanisms; one by demethylating the histone (H3K9me3) of target genes (e.g., *c-Myc* and *MDM2*) and the other by targeting and demethylating a non-histone protein, p53 (p53K372me1). KDM4C activates the expression of the oncogenes c-Myc and MDM2 by removing the repressive methylation (H3K9me3) mark, whereas it inhibits the p53 target gene (e.g., *p21*, *Bax*, and *PUMA*) expression and the p53-induced apoptosis by demethylating p53K372me1. The C-terminal regulatory domain of p53 is crucial for the regulation of its activity and stability. Lysine residues of the p53 C-terminal region are methylated at K370, K372, and K373 by histone lysine methyltransferases (KMT)-3C, KMT1C/D, and KMT5A, respectively^34^. K370me1 and K373me2 repress the transcriptional activity of p53, but K372me1 enhances the activity of p53^35^. Furthermore, increased levels of p53K372me1 elevated the level of p53 acetylated at K381 and K382, which enhances the stability of p53^35^. Importantly, our results demonstrate that KDM4C inhibition enhances the functional activity and stability of p53 by inducing the monomethylation of p53K372, acetylation of p53K381/382, and phosphorylation of p53S15. Hence, our results suggest that p53 at K372me1 is a target site for KDM4C.

In the Cancer Genome Atlas (TCGA) glioblastoma database, Myc expression was upregulated in all glioblastoma subtypes relative to that in the normal human cerebrum^29^. In addition, Myc expression was more increased in recurrent glioblastoma compared to that in newly diagnosed glioblastoma^29^. Although *c-Myc* is infrequently mutated in glioblastoma, increases in its copy number and nuclear staining have been investigated^36^. The expression of Myc core signature^37^ correlates with that of the gene signature in glioblastoma tumorigenicity^29,38^. These results support a relationship between Myc expression and tumorigenicity. Interestingly, several studies have demonstrated that glioblastoma cells overexpress several *KDM* genes, and some KDMs are involved in the Myc-mediated tumorigenesis. c-Myc^39^ and N-Myc^40^ interact with lysine-specific demethylase 1 (LSD1/KDM1A) and promote its recruitment to the E-box chromatin. Furthermore, KDM1A is a cofactor of the repressive function of N-Myc in neuroblastoma. KDM1A also decreases H3K4me3 at the *c-Myc* locus and reduces the expression of Myc^29^ as well as its target genes in glioblastoma cells. Recently, Nagasaka et al.^41^ demonstrated that KDM1A is a direct target of c-Myc. Our findings revealed that KDM4C directly binds to the *c-Myc* P2 and P3 promoters and induces its expression. *KDM4C* and *c-Myc* expressions were positively correlated, whereas those of *KDM4A, KDM4B, and KDM4D* did not correlate with *c-Myc* in glioblastoma. Also, KDM4C has no effects on *N-Myc* expression in glioblastoma (data not shown), and the correlation of the expression between the two genes has not yet been reported. These findings indicate that KDM1A and KDM4C have opposite functions on c-Myc expression and contribute to fine-tuning its expression; KDM1A represses *c-Myc* expression by demethylating the active marker H3K4me2/me1, whereas KDM4C activates its expression by demethylating the repressive marker H3K9me3/me2. Our observation that the inhibition of KDM4C leads to decreased c-Myc expression and increased expression of p53, p21, and PUMA suggests that KDM4C is a novel factor of c-Myc signaling in glioblastoma. Such oncogenic function of KDM4C is caused due to the dissociation of the epigenetic reader heterochromatin protein 1 (HP1) from the target gene promoters, including *c-Myc*, thereby reducing H3K9me3 levels and losing heterochromatin to activate oncogenes and increase genomic instability^12^. c-Myc suppresses the p53 target gene p21^42^ and PUMA^43^ expression by directly binding to the E-box promoter, whereas p53 binds to the c-Myc NHE promoter and represses its expression^44^. Therefore, the reduction of c-Myc by KDM4C inhibition increases p21 and PUMA levels, together with p53 activation. Consequently, KDM4C inhibition might synergistically increase the levels of p21 and PUMA expression. Because of glioblastoma’s addiction to Myc signaling^45,46^, Myc proteins are considered as suitable therapeutic targets. However, Myc protein has long been considered as an undruggable cancer target, and a clinically available direct inhibitor of Myc has not been developed due to the lack of deep surface-binding pockets^47^. As an alternative strategy, Myc inhibition can be achieved by epigenetic silencing of *Myc* genes (e.g., bromodomain inhibitor) or by blocking the signaling pathways downstream of the Myc transcription factor. These results suggest that the KDM4C-cMyc-p53 signaling cascade is a novel mechanism underlying the progression of glioblastoma. Therefore, targeting KDM4C as an indirect Myc-targeting approach may be a promising novel cancer therapy for glioblastoma.

## Supplementary information

Supplementary data

Supplementary figures
