# Supplementary material for: Histone demethylase KDM4C controls tumorigenesis of glioblastoma by epigenetically regulating p53 and c-Myc
Source: Cell Death Dis. 2021 Jan 18;12(1):89. doi: 10.1038/s41419-020-03380-2 (PMC7814060; doi:10.1038/s41419-020-03380-2)
Supplement: Supplementary file 1 — Supplementary data [file 41419_2020_3380_MOESM1_ESM.doc]

**Supplementary materials and methods**

**Histone demethylase KDM4C controls tumorigenesis of glioblastoma by epigenetically regulating p53 and c-Myc**

**Authors:** Dong Hoon Lee^1^, Go Woon Kim^1^, Jung Yoo^1^, Sang Wu Lee^1^, Yu Hyun Jeon^1^, So Yeon Kim^1^, Hyeok Gu Kang ^2,3^, Da-Hyun Kim^2,3^, Kyung-Hee Chun^2,3^, Junjeong Choi^1^, and So Hee Kwon^1*^

**Affiliations:**

^1^College of Pharmacy, Yonsei Institute of Pharmaceutical Sciences, Yonsei University, Incheon, 21983, Republic of Korea;

^2^Department of Biochemistry and Molecular Biology, Yonsei University College of Medicine, Seoul, 03722, Republic of Korea

^3^Brain Korea 21 PLUS Project for Medical Science, Yonsei University College of Medicine, Seoul, 03722, Republic of Korea

*Corresponding author:

So Hee Kwon: soheekwon@yonsei.ac.kr

# Supplementary tables

# Supplementary table 1. Antibodies

| **Reagent or resource** | **Source** | **Identifier** |
| --- | --- | --- |
| Acetylated α-tubulin | Sigma | T6793, 1:2000 |
| β-Actin | Santa Cruz Biotechnology | sc-47778, 1:1000 |
| Bak | Santa Cruz Biotechnology | sc-832, 1:1000 |
| Cdk2 | Santa Cruz Biotechnology | sc-6248, 1:1000 |
| Cdk4 | Santa Cruz Biotechnology | sc-23896, 1:1000 |
| Cdk6 | Santa Cruz Biotechnology | sc-7961, 1:1000 |
| Cyclin A1 | Santa Cruz Biotechnology | sc-239, 1:500 |
| Cyclin D1 | Santa Cruz Biotechnology | sc-718, 1:500 |
| Cyclin E2 | Santa Cruz Biotechnology | sc-28351, 1:2000 |
| GST | Santa Cruz Biotechnology | sc-459, 1:1000 |
| His | Santa Cruz Biotechnology | sc-8036, 1:1000 |
| MDM2 | Santa Cruz Biotechnology | sc-965, 1:500 |
| C-Myc | Santa Cruz Biotechnology | sc-40, 1:500 |
| N-Myc | Santa Cruz Biotechnology | sc-142, 1:500 |
| PUMA | Santa Cruz Biotechnology | sc-28226, 1:500 |
| α-tubulin | Santa Cruz Biotechnology | sc-32293, 1:2000 |
| p53 | Santa Cruz Biotechnology | sc-126, 1:1000 |
| p21 | Santa Cruz Biotechnology | sc-759, 1:500 |
| p27 | Santa Cruz Biotechnology | sc-543, 1:500 |
| Cyclin D2 | Millipore | 3741T, 1:500 |
| Histone H3 | Millipore | 06-755, 1:500 |
| H3K4me3 | Millipore | 07-473, 1:1000 |
| KDM4A | Millipore | MABE222, 1:1000 |
| Bax | Cell Signaling Technology | 2772, 1:1000 |
| Bcl-xL | Cell Signaling Technology | 2762, 1:500 |
| Caspase-3 | Cell Signaling Technology | 9662, 1:1000 |
| Cyclin D2 | Cell Signaling Technology | 3741, 1:500 |
| GAPDH | Cell Signaling Technology | 3693, 1:2000 |
| Phospho-p53 (Ser15) | Cell Signaling Technology | 9284, 1:500 |
| Acetyl-p53 (Lys382) | Cell Signaling Technology | 2525, 1:500 |
| H3K9me1 | Abcam | ab9045, 1:500 |
| H3K9me2 | Abcam | ab1220, 1:500 |
| H3K9me3 | Abcam | ab8898, 1:500 |
| KDM4B | Abcam | ab91549, 1:500 |
| KDM4C | Abcam | ab85454, 1:1000 |
| KDM4D | Abcam | ab63199, 1:1000 |
| MDMX | Abcam | ab154324, 1:500 |
| p53K372me1 | Abcam | ab10633, 1:1000 |
| PARP | BD Bioscience | 551024, 1:500 |
| XIAP | BD Bioscience | 10716, 1:1000 |
| HA | Roche | 11867423001, 1:1000 |
| Acetyl-p53 (Lys372) | St John’s laboratory | STJ90151, 1:500 |
| Acetyl-p53 (Lys381) | St John’s laboratory | STJ90131, 1:500 |

# Supplementary table 2. DNA constructs

| **Plasmid name** | **Characteristics** | **Source** |
| --- | --- | --- |
| pCMV-HA-KDM4A | HA-tagged expressing plasmid | Addgene #24180 |
| pCMV-HA-KDM4C | HA-tagged expressing plasmid | Addgene #24214 |
| pCMV-HA-KDM4C H190A | Catalytic dead mutant | This work |
| pGEX-4T-1 KDM4C 1-352 | GST-tagged expressing plasmid | This work |
| pGEX-6P-1 KDM4C 280-720 | GST-tagged expressing plasmid | This work |
| pGEX-4T-1 KDM4C 660-1058 | GST-tagged expressing plasmid | This work |
| pDEST15-GST-p53 | GST-tagged expressing plasmid | This work |
| pGEX-4T-1 p53 1-83 | GST-tagged expressing plasmid | This work |
| pGEX-4T-1 p53 89-289 | GST-tagged expressing plasmid | This work |
| pGEX-4T-1 p53 290-393 | GST-tagged expressing plasmid | This work |
| pcDNA-HA-p53 | HA-tagged expressing plasmid | This work |
| pgl2-basic-p21 | Luciferase reporter assay plasmid | This work |
| pgl2-basic-bax | Luciferase reporter assay plasmid | This work |
| pcDNA-c-MYC | c-MYC expressing plasmid | Addgene #16011 |

# Supplementary table 3. qRT-PCR primers

| **Gene** | **Primer sequence** |
| --- | --- |
| p21 | Forward: CACCGAGACACCACTGGAGG  Reverse: GAGAAGATCAGCCGGCGTTT |
| MDM2 | Forward: ATCTTGGCCAGTATATTATG  Reverse: GTTCCTGTAGATCATGGTAT |
| PUMA | Forward: GACCTCAACGCACAGTA  Reverse: CTAATTGGGCTCCATCT |
| p53 | Forward: GAGGGATGTTTGGGAGATGTAAGAAATG  Reverse: TTCACAGATATGGGCCTTGAAGTTAGAGAA |
| Bax | Forward: TCTACTTTGCCAGCAAACTGG  Reverse: TGTCCAGCCCATGATGGTTCT |
| GADD45 | Forward: TGCGAGAACGACATCAACAT  Reverse: TCCCGGCAAAAACAAATAAG |
| GAPDH | Forward: CATGAGAAGTATGACAACAGCCT  Reverse: AGTCCTTCCACGATACCAAAGT |
| KDM4A | Forward: CCTCACTGCGCTGTCTGTAT  Reverse: CCAGTCGAAGTGAAGCACAT |
| KDM4B | Forward: CGGGTTCTATCTTTGTTTCTCTCACCCG  Reverse: AAGGAAGCCTCTGGAACACCTG |
| KDM4C | Forward: GGTCAACCCCAACGTGAAGT  Reverse: CGTTTGACCCACGGAAATG |
| KDM4D | Forward: CGGGATCTGCACAGATTATCCACCCG  Reverse: AGTTTCTGAGGAGGGCGACCA |
| KDM3A | Forward: ATGCCCACACAGATCATTCC  Reverse: CTGCACCAAGAGTCGGTTTT |
| c-MYC | Forward: CCCCCGAATTGTTTTCTCTT  Reverse: TCTCATCCTTGGTCCCTCAC |

# Supplementary methods

# GST pull-down assay

Recombinant GST-fusion protein and His-fusion protein or recombinant HA-KDM4C were mixed in the buffer A (50 mM HEPES pH 7.9, 300 mM NaCl, 2 mM MgCl_2_, 0.5 mM EDTA, 0.05% Triton X-100, 10% glycerol, 1 mM PMSF, and 0.1 mg/ml BSA). The mixture was incubated with GST or HA antibody and protein A/G beads at 4°C for overnight. Beads mixtures were washed three times using buffer A and eluted by boiling in 5X sample buffer for SDS-PAGE. The eluate and 5% of input were analyzed by western blot using anti-GST, anti-His, and anti-HA antibodies.

# Data Mining

To study the gene expression profile of glioblastoma, we retrieved a DNA microarray dataset numbered GSE90886 and GSE36245 from Gene Expression Omnibus. In GSE90886 dataset, microarray data on Affymetrix Human Genome Expression array were derived from tissues of normal or glioblastoma patients. Differentially expressed genes (DEGs) were identified between glioblastoma samples and normal samples using the Prism program. ** *P* < 0.01 versus the normal samples, Nonparametric test (Mann-Whitney U test). In GSE36245 dataset, microarray data on Affymetrix Human Genome U133 Plus 2.0 Array were derived from glioblastoma patient samples. Correlation of KDM4C and c-Myc was done using Spearman Correlation Analysis after downloading data from microarray datasets.
