# Supplementary material for: Histone demethylase KDM4C controls tumorigenesis of glioblastoma by epigenetically regulating p53 and c-Myc
Source: Cell Death Dis. 2021 Jan 18;12(1):89. doi: 10.1038/s41419-020-03380-2 (PMC7814060; doi:10.1038/s41419-020-03380-2)
Supplement: Supplementary file 2 — Supplementary figures [file 41419_2020_3380_MOESM2_ESM.pdf]

Supplementary Figures

Histone demethylase KDM4C controls tumorigenesis of glioblastoma by epigenetically regulating p53 and c-Myc

Dong Hoon Lee[1], Go Woon Kim[1], Jung Yoo[1], Sang Wu Lee[1], Yu Hyun Jeon[1], So Yeon Kim[1], Hyeok Gu Kang [2,3], Da-Hyun Kim[2,3], Kyung-Hee Chun[2,3], Junjeong Choi[1] and So Hee Kwon[1*]

[1]College of Pharmacy, Yonsei Institute of Pharmaceutical Sciences, Yonsei University, Incheon, 21983, Republic of Korea;

[2]Department of Biochemistry and Molecular Biology, Yonsei University College of Medicine, Seoul, 03722, Republic of Korea

[3]Brain Korea 21 PLUS Project for Medical Science, Yonsei University College of Medicine, Seoul, 03722, Republic of Korea

*Corresponding author:

So Hee Kwon: soheekwon@yonsei.ac.kr

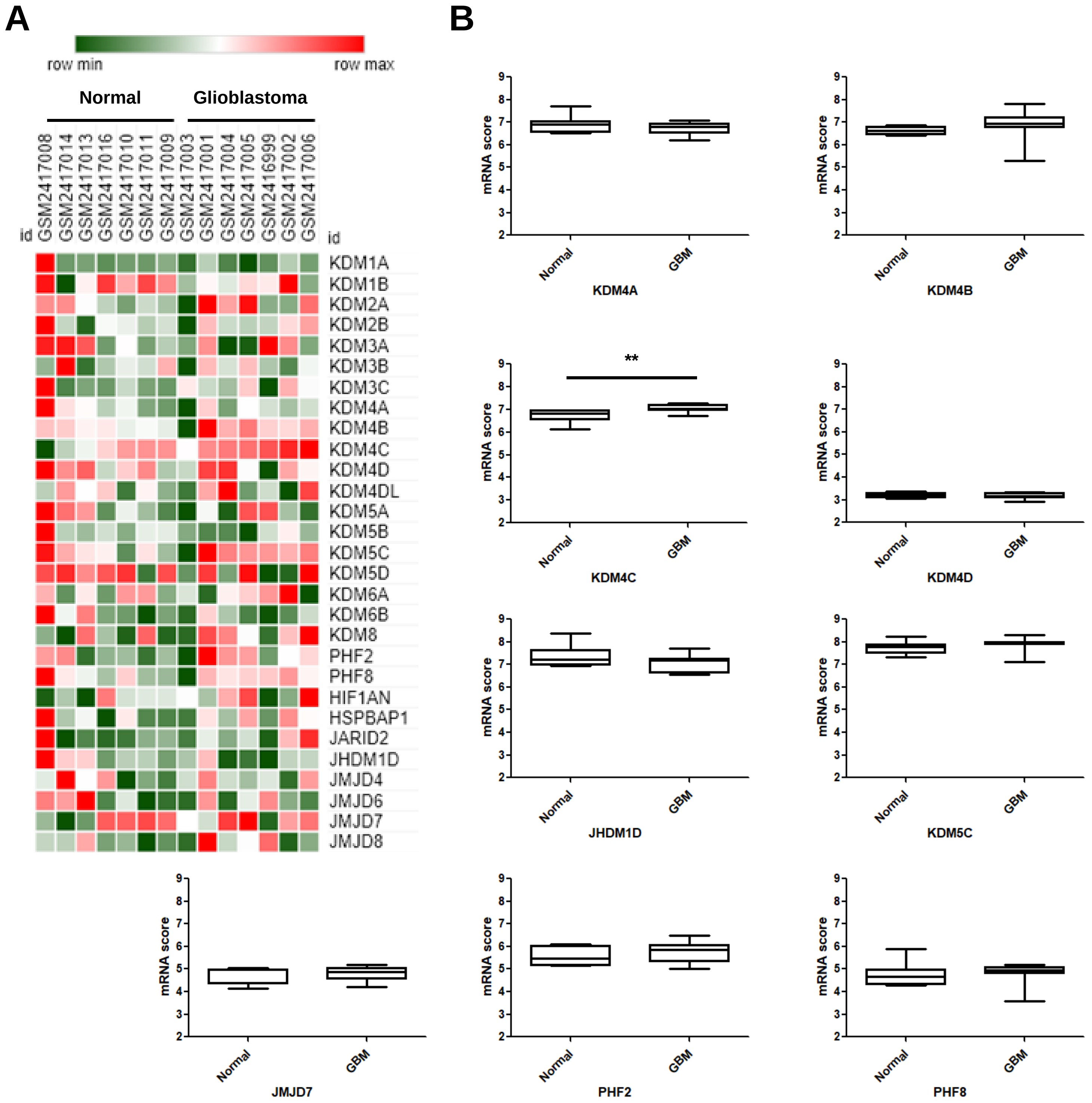

Fig. S1 Microarray database analysis identifies differentially expressed genes in patients with glioblastoma.

(A) Heatmap representation of the expression patterns of 29 KDMs in GEO dataset GSE90886 (GPL15207, glioblastoma tissue *n* = 7 and normal brain tissue *n* = 7). The Heatmap was drawn using the web site (https://software.broadinstitute.org/morpheus). (B) Glioblastoma patient tumors showed higher expression scores of KDM4C than normal tissue samples. The y axis describes $\log_2$ normalized expression scores. $^{**}P < 0.01$ *vs.* the normal samples, Nonparametric test (Mann-Whitney U test).

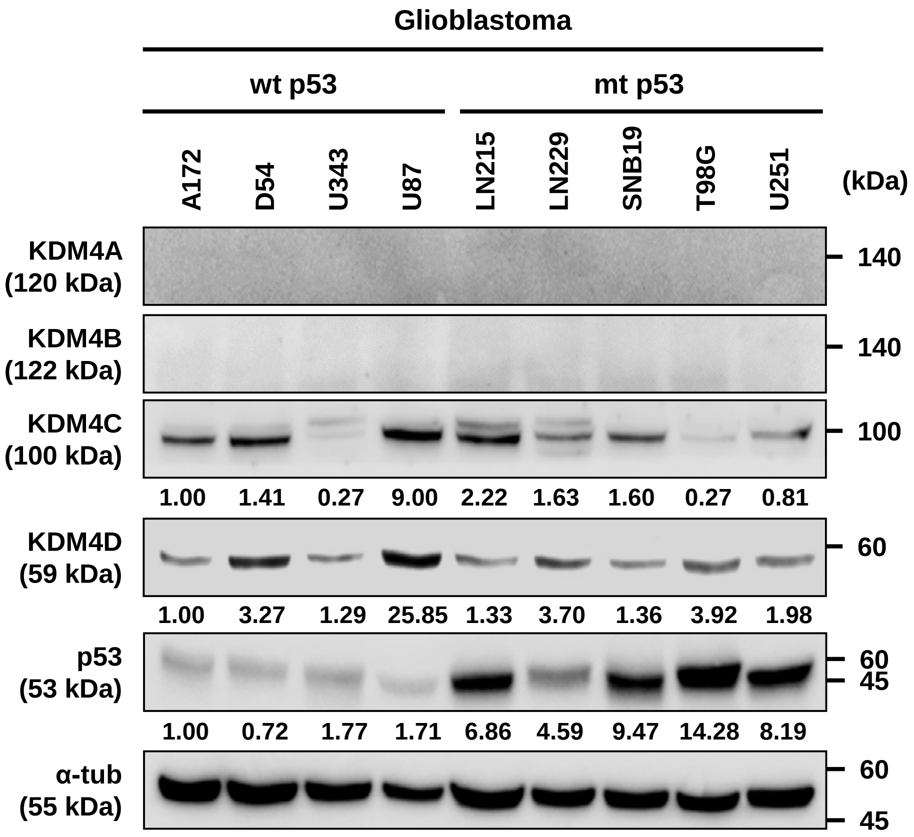

Fig. S2 KDM4C is highly expressed in glioblastoma cells.
Immunoblot analysis of KDM4 and p53 protein expression in 9 glioblastoma cell lines. α-Tubulin was used as an equal loading control. The levels of KDM4 and p53 were quantified against α-tubulin, and control levels were set at 1.

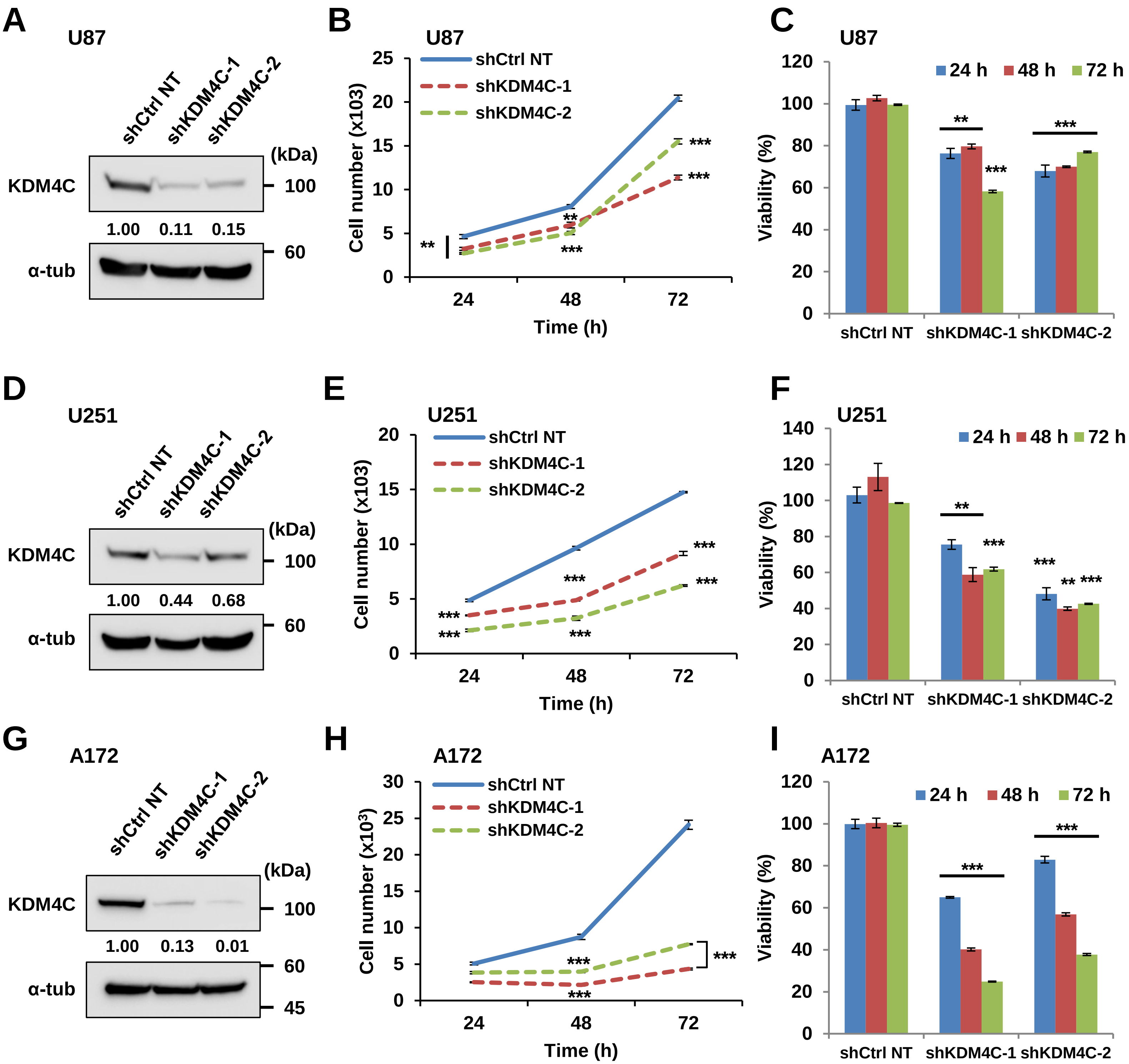

Fig. S3 Depletion of KDM4C inhibits glioblastoma cell growth and viability. Immunoblotting of KDM4C expression in U87 (A), U251 (D), and A172 (G) cells. α-Tubulin was used as a loading control. KDM4C expression levels were quantified against α-tubulin and control levels were set at 1. Cell growth and viability assay of U87 (B-C), U251 (E-F), and A172 (H-I) with KDM4C knockdown using shRNA. Knockdown of KDM4C in glioblastoma by expressing shRNA constructs targeting KDM4C (shKDM4C) or a non-target control shRNA (shCtrl NT). Error bars represent SD (n = 3). ** P < 0.01 and *** P < 0.001 *vs*. shCtrl NT; Student's t-tests.

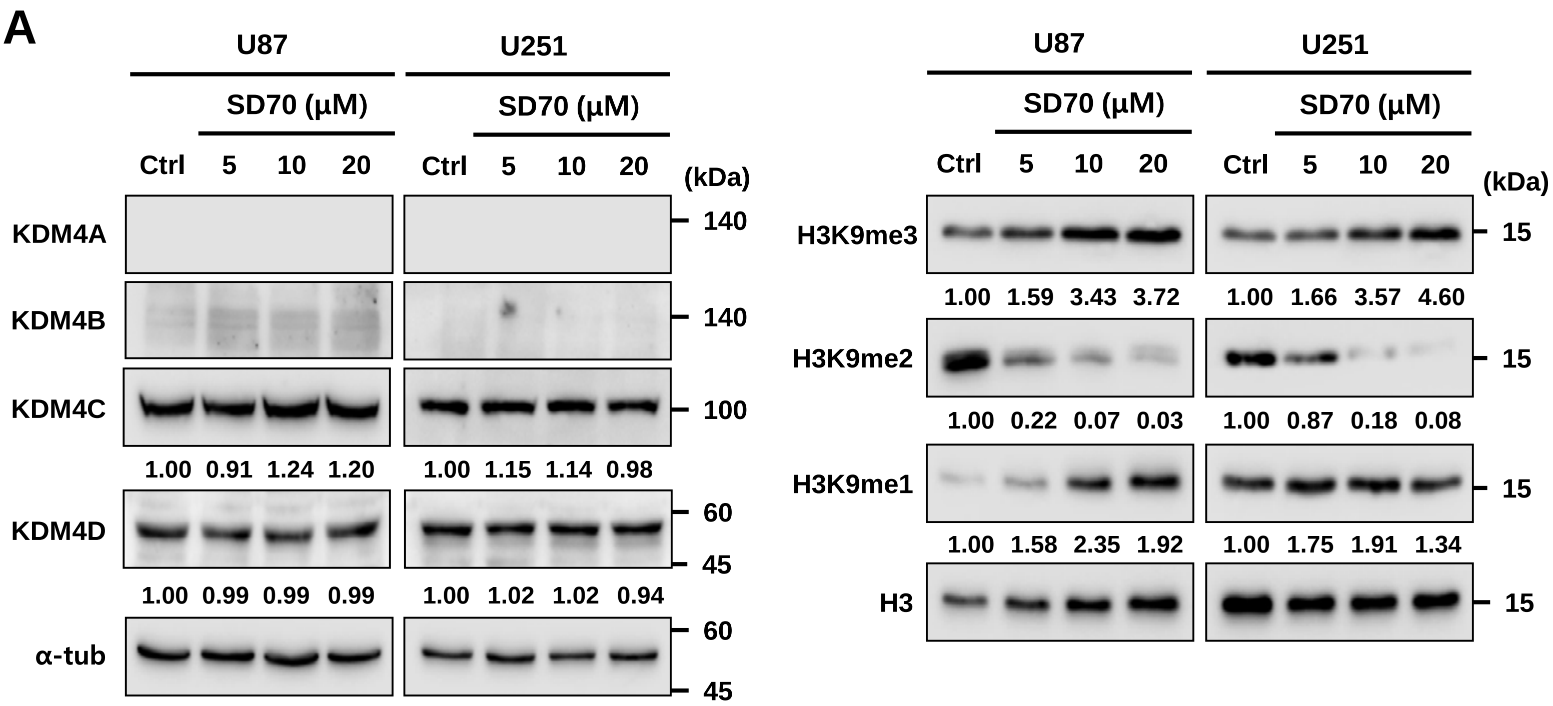

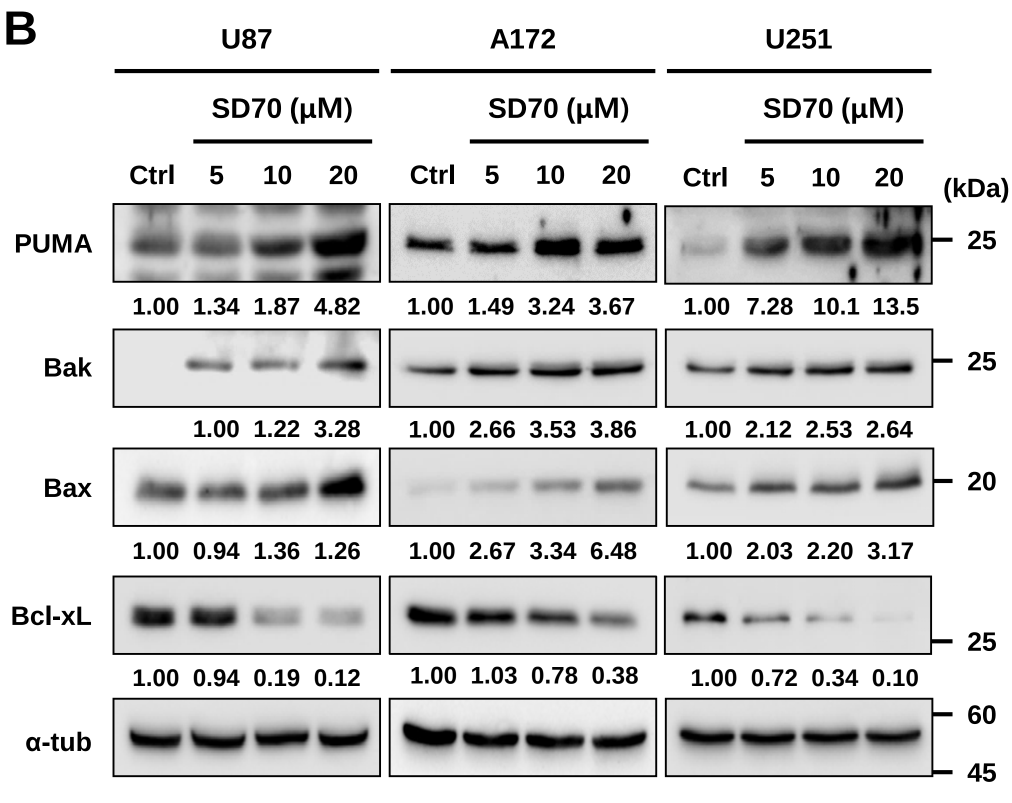

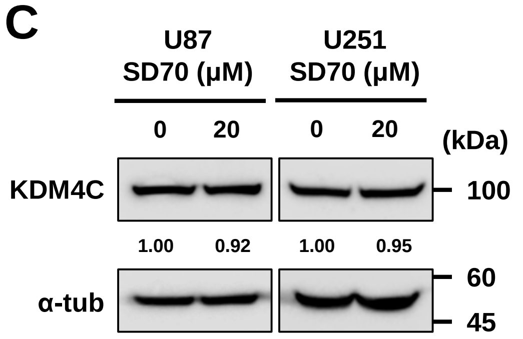

D

Cell line (72h)	SD70 (μM)	
	GI_{50}	IC_{50}
**U87**	1.752	2.176
**A172**	1.302	1.302
**U251**	2.132	2.649
**LN215**	1.979	1.863
**T98G**	1.808	1.712

Fig. S4 KDM4C inhibition triggers apoptosis in glioblastoma cells.
(A-B) Immunoblot analysis of KDM4s, histones, apoptotic proteins in U87, A172, and U251 cells treated with 0.1% DMSO (control) or SD70 at the indicated concentrations for 24 h. α-Tubulin and histone H3 were used as loading controls. Protein expression levels were quantified against α-tubulin or H3, and control levels were set at 1. (C) Immunoblot analysis of KDM4C treated with SD70. (D) GI_{50} and IC_{50} value of SD70 in glioblastoma cell lines. Data present mean ± SD (*n* = 3).

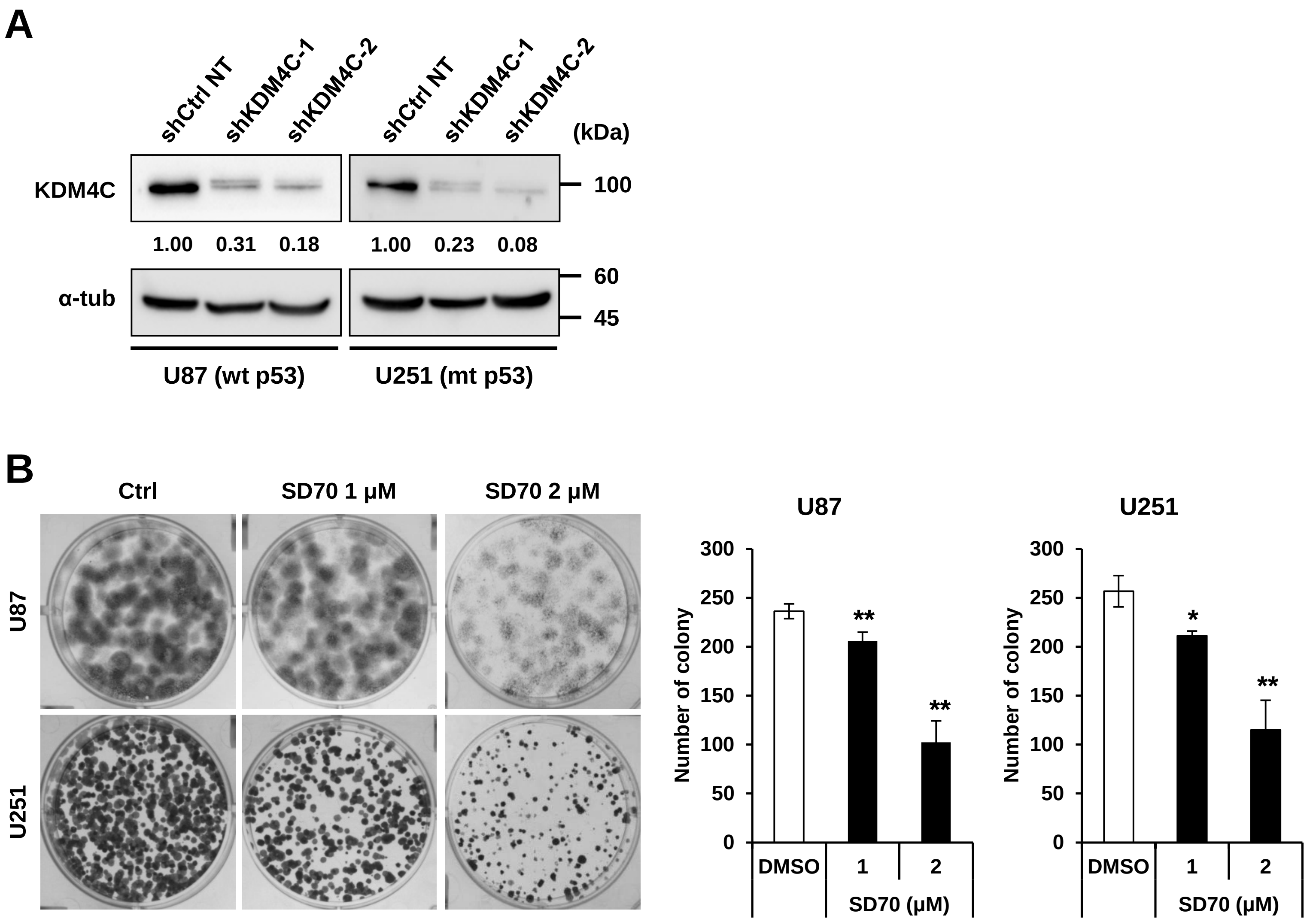

Fig. S5 Selective inhibition of KDM4C blocks proliferation of glioblastoma cells.
(A) Immunoblot analysis of KDM4C in shCtrl NT and shKDM4C expressing U87 and U251 cells. α-Tubulin was used as a loading control. KDM4C expression levels were quantified against α-tubulin, and control levels were set at 1. (B) Inhibition of cell proliferation was validated in liquid colony formation assays in SD70 treated glioblastoma cells. Representative 6 well plates images for U87 and U251 were shown. The right panels were generated by counting stained liquid colony formation assays in glioblastoma cells with SD70 treatment. Data present mean ± SD from three independent experiments. *P < 0.05 and **P < 0.01 *vs*. the control group; Student's *t*-tests.

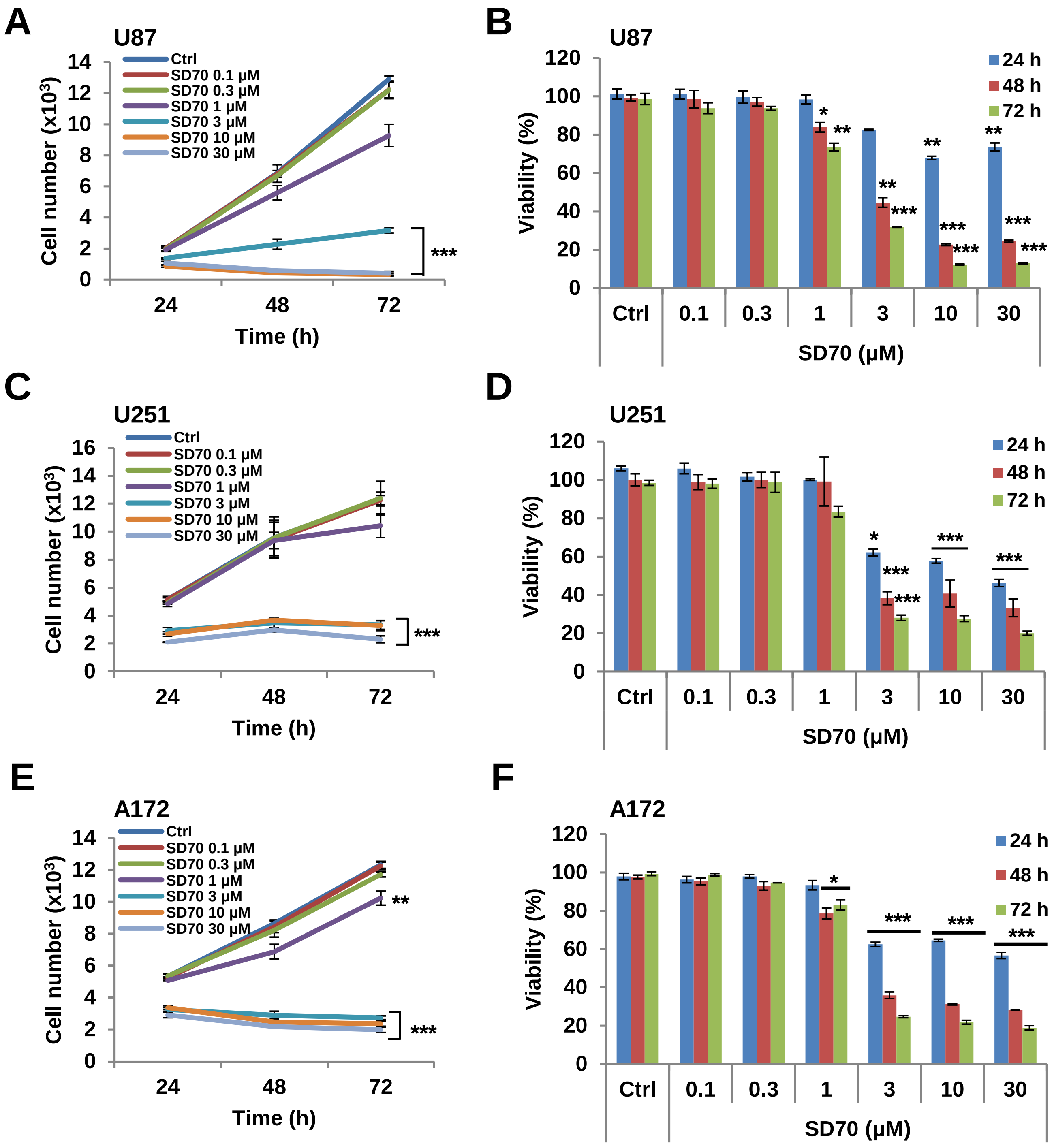
A
U87
Ctrl
SD70 0.1 μM
SD70 0.3 μM
SD70 1 μM
SD70 3 μM
SD70 10 μM
SD70 30 μM
Cell number (x10³)
Time (h)
***
B
U87
Viability (%)
24 h
48 h
72 h
Ctrl
SD70 (μM)
C
U251
D
U251
E
A172
F
A172

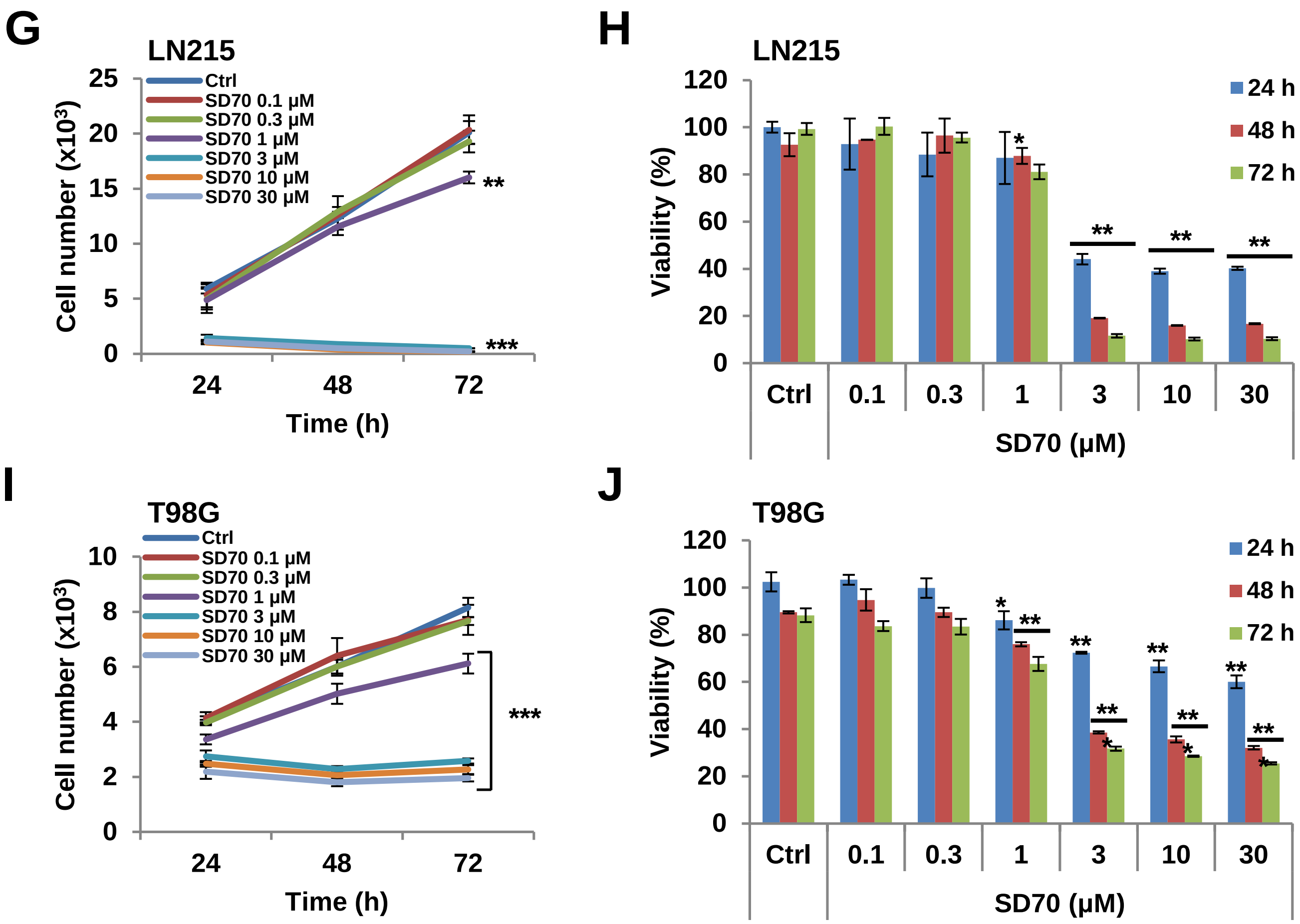

Fig. S6 Inhibition of KDM4C suppresses glioblastoma cell growth and viability.
Cell growth and viability of U87 (A, B), U251 (C, D), A172 (E, F), LN215 (G, H) and T98G (I, J) cells cultured with 0.1% DMSO (control) or SD70 at the indicated concentrations for 72 h. Viable cell numbers and viability were measured using CCK8 assays. Cell counts were estimated indirectly from a standard curve generated using solutions of known cell counts. Absorbance was normalized to that of the negative control at each time interval. Data present mean ± SD from three independent experiments. **P* < 0.05, ***P* < 0.01, and ****P* < 0.001 *vs*. control group; Student's *t*-tests.

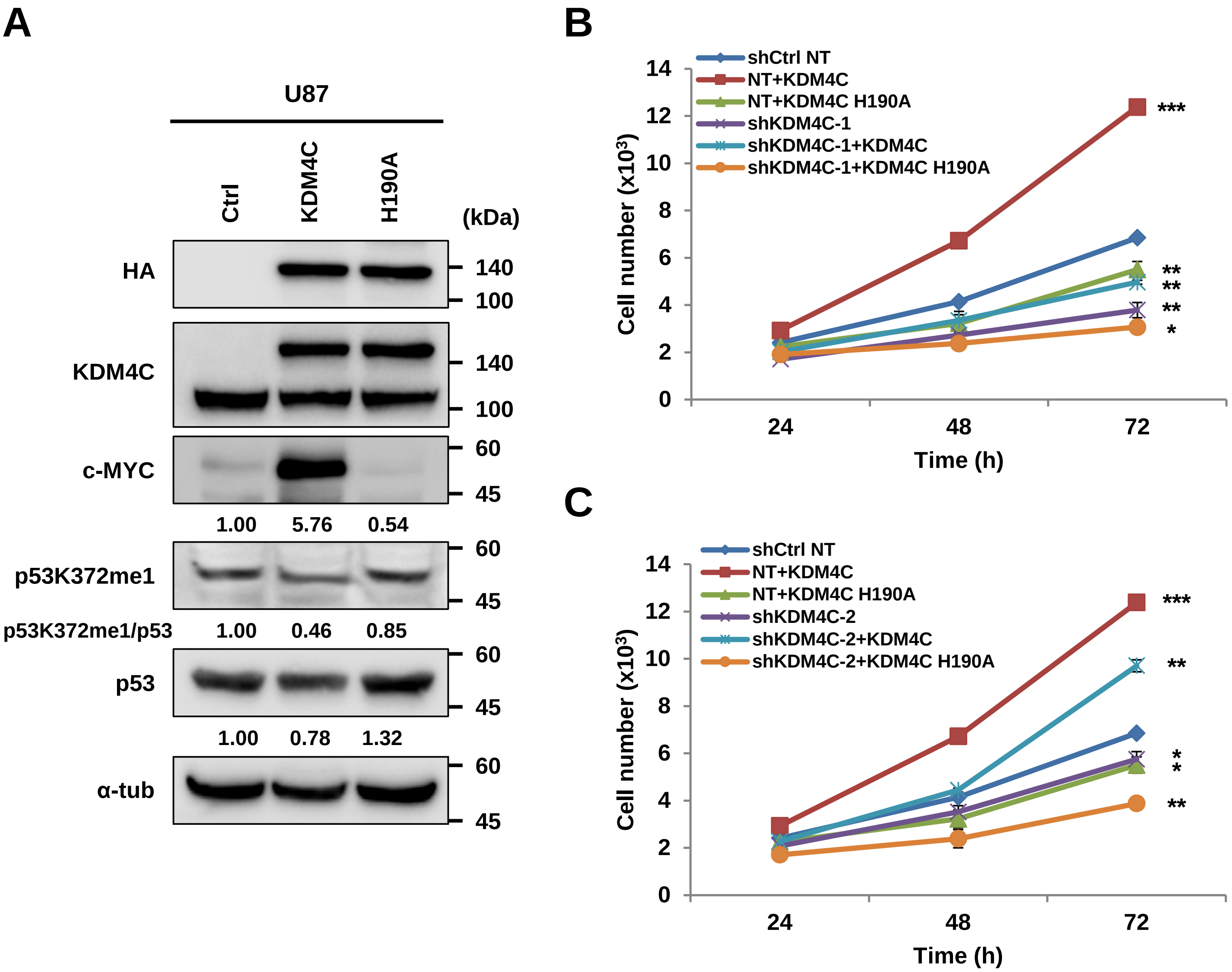

Fig. S7 The enzymatic activity of KDM4C is essential for glioblastoma cell growth and viability.

(A) Immunoblot analysis of KDM4C, c-Myc, and p53 in U87 cells transfected with empty vector, HA-KDM4C, or HA-KDM4C H190A. α-Tubulin was used as a loading control. Protein expression levels were quantified against α-tubulin and control levels were set at 1. (B-C) Cell growth and viability of U87 cells expressing shCtrl NT or shKDM4C followed by transfection with empty vector, HA-KDM4C, or HA-KDM4C H190A for 72 h. Viable cell numbers and viability were measured using CCK8 assays. Cell counts were estimated indirectly from a standard curve generated using solutions of known cell counts. Absorbance was normalized to that of the negative control at each time interval. Data present mean ± SD from three independent experiments. *$P < 0.05$, **$P < 0.01$, and ***$P < 0.001$ *vs.* control group; Student's *t*-tests. shCtrl NT or shKDM4C.

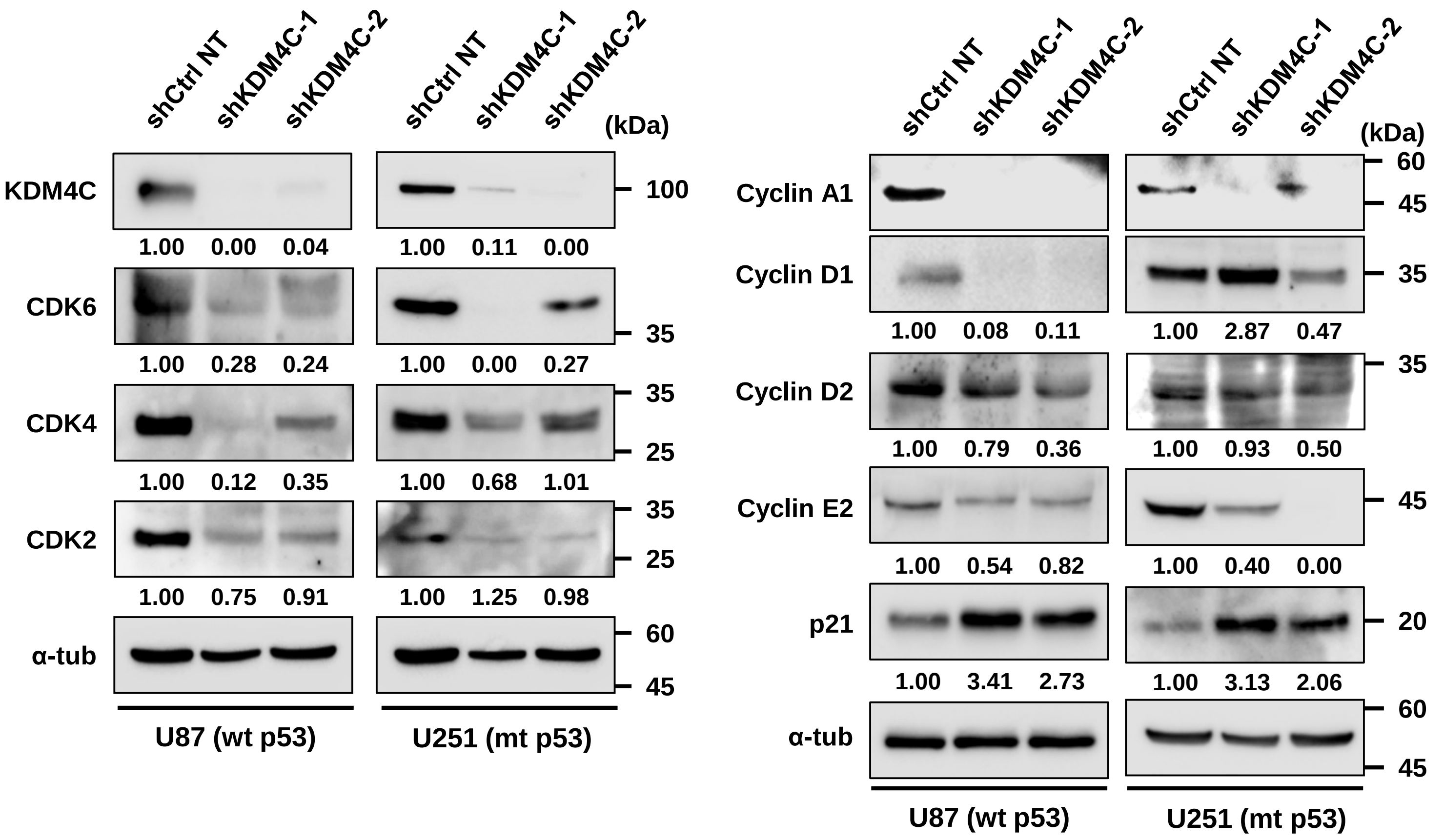

Fig. S8 KDM4C knockdown reduces cell cycle proteins in glioblastoma cells.
Immunoblot analysis of KDM4C, CDKs, cyclins, and p21 in shCtrl NT and shKDM4C expressing U87 and U251 cells. α-Tubulin was used as a loading control. Protein expression levels were quantified against α-tubulin, and control levels were set at 1.

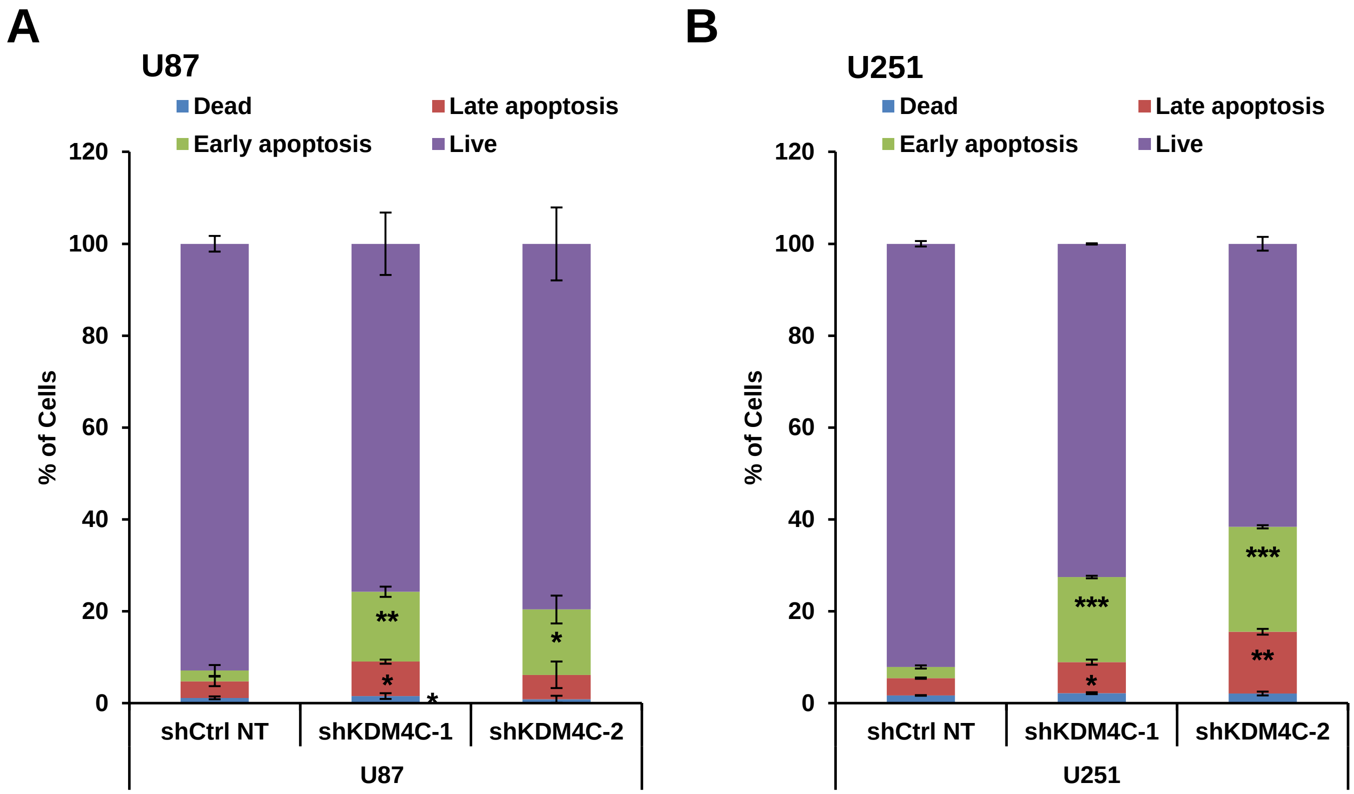

Fig. S9 KDM4C knockdown triggers apoptosis in glioblastoma cells.
(A-B) Apoptosis analysis of shCtrl NT and shKDM4C expressing U87 (A) and U251 (B) cells as determined by flow cytometry. Data present mean ± SD from three independent experiments. *$P < 0.05$, **$P < 0.01$, and ***$P < 0.001$ *vs.* shCtrl NT; Student's *t*-tests.

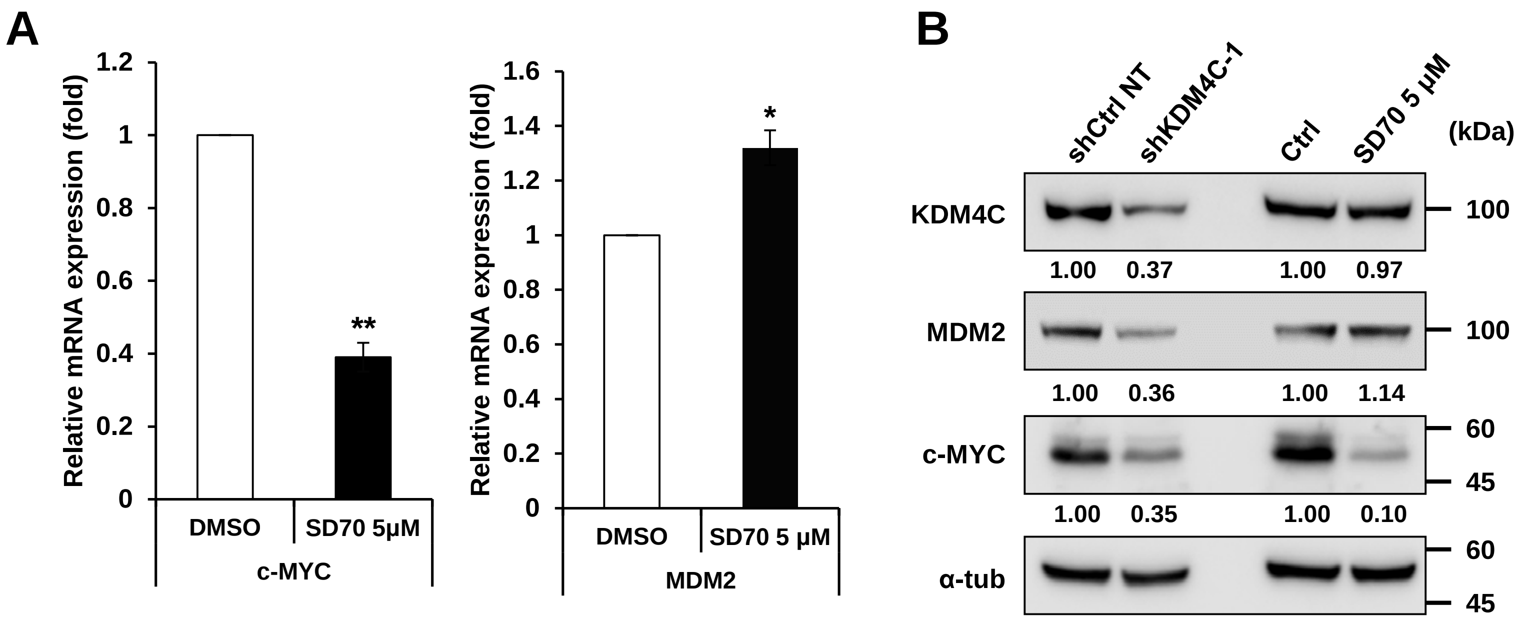

Fig. S10 Inhibition of KDM4C reduces the expression levels of c-Myc and MDM2.
(A) RT-qPCR showing c-Myc and MDM2 mRNA levels in U87 cells treated with 0.1% DMSO (control) or SD70 (5 μM). mRNA expression levels were normalized to GAPDH mRNA levels. (B) Immunoblot analysis of KDM4C, c-Myc, and MDM2 expression in U87 and U251 cells treated with shRNA or SD70 (5 μM) for 24 h. α-Tubulin was used as an equal loading control. Protein expression levels were quantified against α-tubulin, and control levels were set at 1.

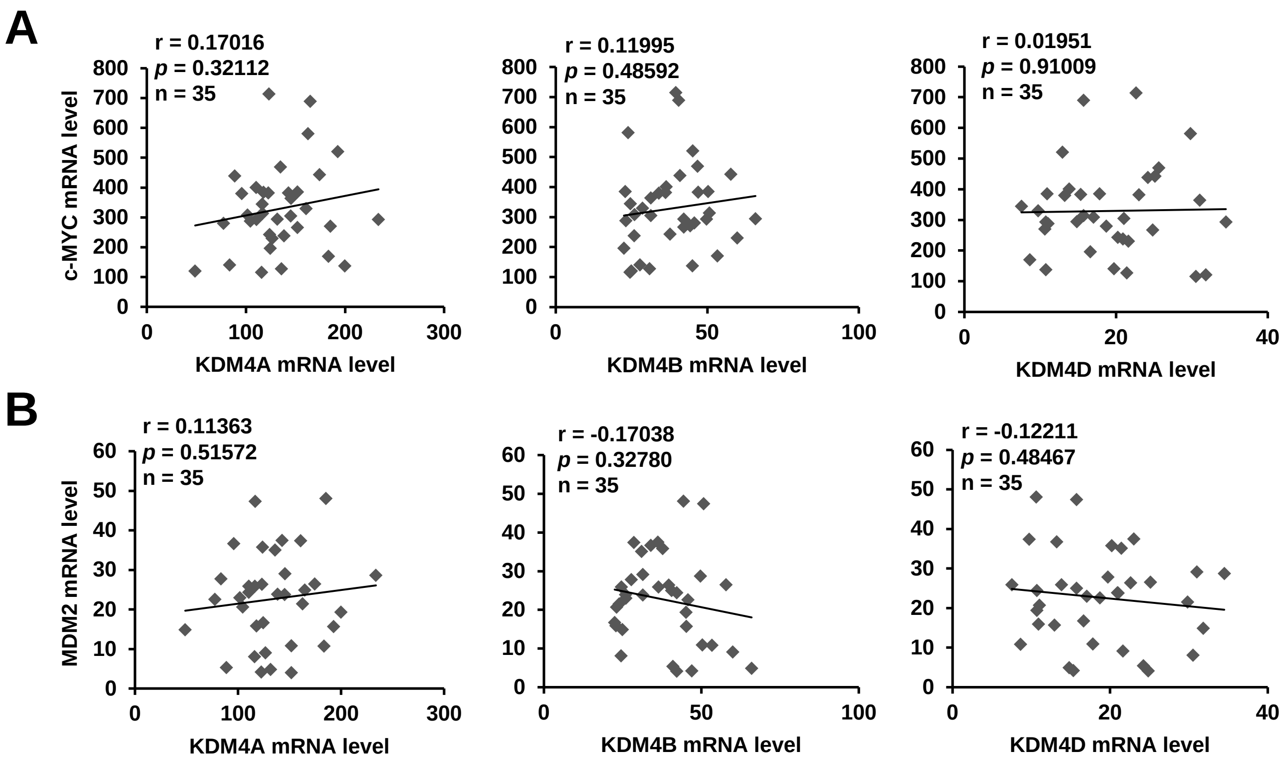

Fig. S11 Correlation analysis between KDM4s, c-Myc, and MDM2 expressions.

(A-B) GEO analysis of microarray dataset GSE36245 (GPL570, glioblastoma brain tumors, *n* = 35). Pearson correlation was used to measure the relationship between KDM4A, KDM4B, KDM4D and c-Myc (A) and MDM2 (B) mRNA levels. Individual graph showed mRNA expression levels for KDM4 and other genes in glioblastoma tumor samples, in which each dot corresponds to one tumor sample.

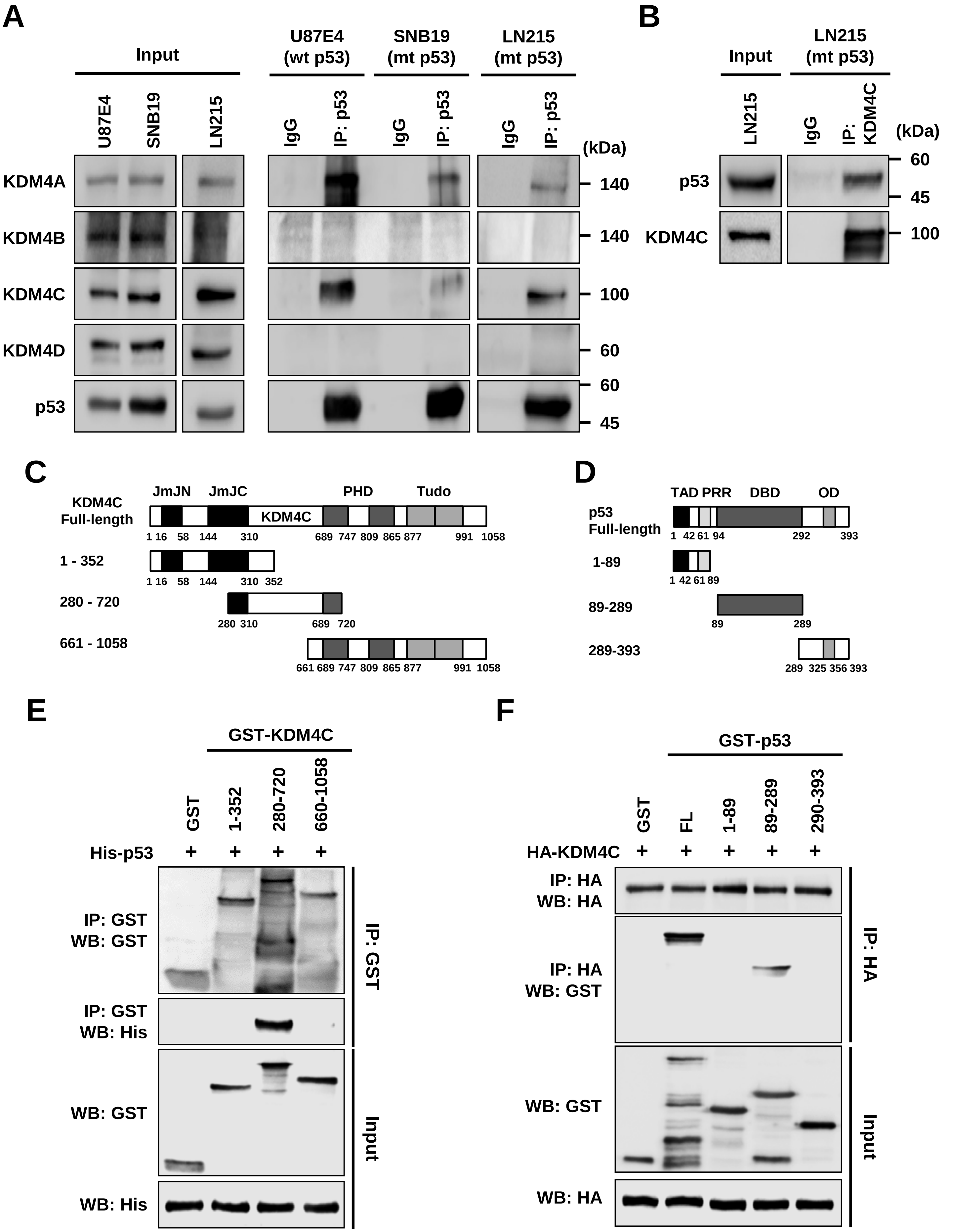
A
Input
U87E4
SNB19
LN215
U87E4 (wt p53)
SNB19 (mt p53)
LN215 (mt p53)
IgG
IP: p53
(kDa)
KDM4A
KDM4B
KDM4C
KDM4D
p53
140
140
100
60
60
45
B
Input
LN215 (mt p53)
LN215
IgG
IP: KDM4C
(kDa)
p53
KDM4C
60
45
100
C
KDM4C Full-length
JmJN
JmJC
KDM4C
PHD
Tudo
1 16 58 144 310 689 747 809 865 877 991 1058
1 - 352
1 16 58 144 310 352
280 - 720
280 310 689 720
661 - 1058
661 689 747 809 865 877 991 1058
D
p53 Full-length
TAD PRR DBD OD
1 42 61 94 292 393
1-89
1 42 61 89
89-289
89 289
289-393
289 325 356 393
E
GST-KDM4C
GST
1-352
280-720
660-1058
His-p53
+ + + +
IP: GST
WB: GST
IP: GST
WB: His
WB: GST
WB: His
IP: GST
Input
F
GST-p53
GST
FL
1-89
89-289
290-393
HA-KDM4C
+ + + + +
IP: HA
WB: HA
IP: HA
WB: GST
WB: GST
WB: HA
IP: HA
Input

Fig. S12 KDM4C and KDM4A interact with p53.

(A) Complex formation of endogenous KDM4 and p53 in U87E4, LN215, and SNB19 glioblastoma cells. The interaction was measured by immunoprecipitation with an anti-p53 antibody, followed by immunoblotting with antibodies as indicated (*n* = 3). 10% of total cell lysates used in immunoprecipitation are shown as input. (B) The interaction was measured by immunoprecipitation with an anti-KDM4C antibody, followed by immunoblotting with antibodies as indicated (*n* = 3). (C) Schematic representation of the GST-tagged KDM4 deletion constructs and (D) GST-tagged p53 truncated mutants used in this study. (E-F) GST pull-down assay. One representative experiment is presented (*n* = 3). (E) KDM4C interacts with p53 through the middle domains. Indicated deletion derivatives of GST-KDM4C were examined for binding to recombinant His-p53. (F) p53 interacts with KDM4C through the DBD domains. Indicated deletion derivatives of GST-p53 were examined for binding to recombinant HA-KDM4C. HA-KDM4C proteins were incubated with glutathione agarose loaded with GST alone or a GST-p53 fusion protein. After washing, bound proteins were run on SDS-PAGE, and retained p53 was detected by western blotting using indicated antibodies.

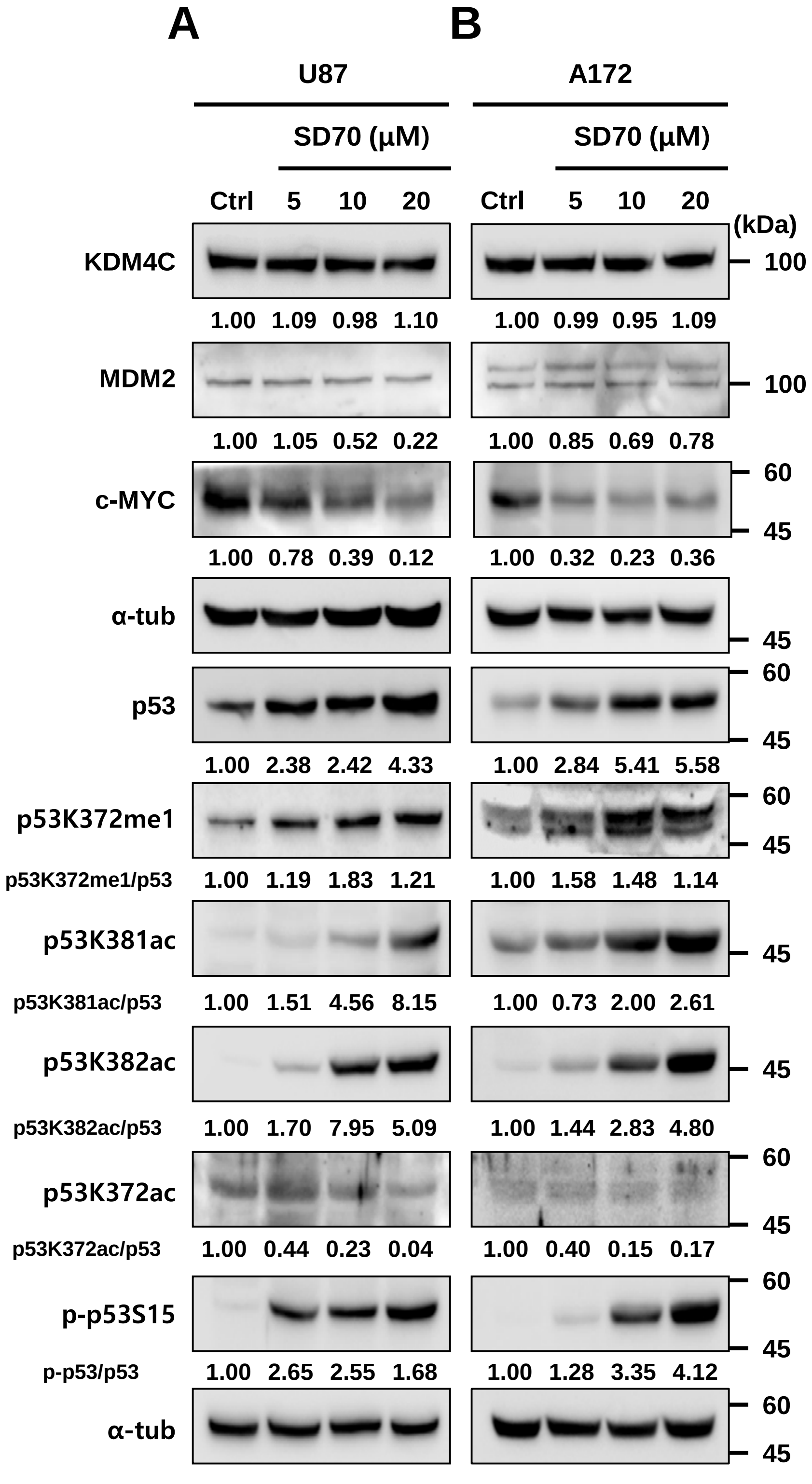

Fig. S13 Inhibition of KDM4C reduces the expression levels of c-Myc and MDM2 and alters posttranslational modifications of p53 in glioblastoma cells.

(A-B) Immunoblot analysis of indicated proteins expression in U87 (A) and A172 (B) cells treated with 0.1% DMSO (control) or SD70 (5, 10, 20 μM) for 24 h. α-Tubulin was used as an equal loading control. Protein expression levels were quantified against α-tubulin, and control levels were set at 1. The modification levels of p53 was quantified relative to p53.

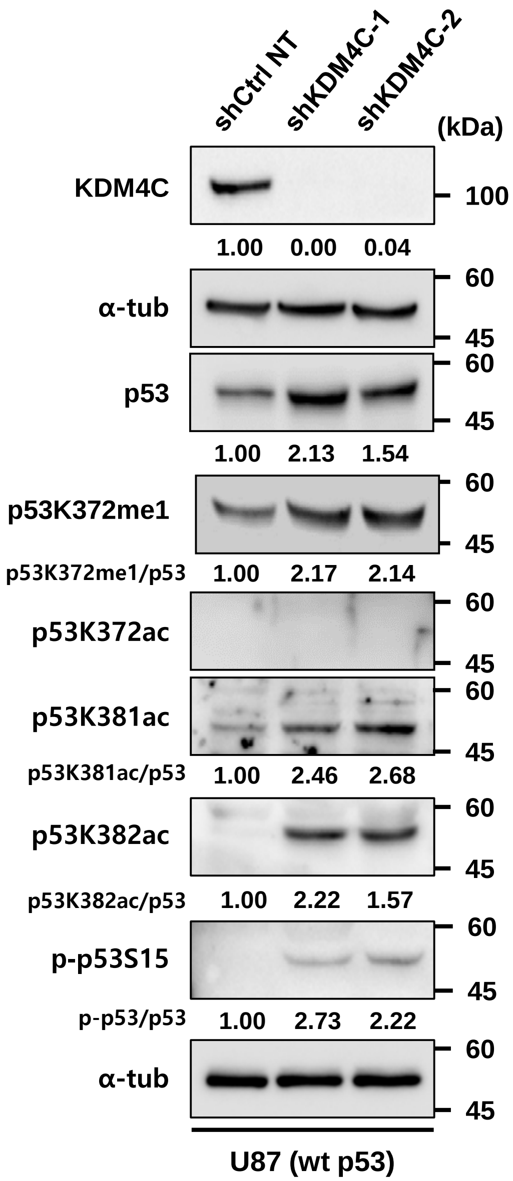

Fig. S14 KDM4C knockdown alters posttranslational modifications of p53.
Immunoblot analysis of indicated proteins expression and p53 modifications in shCtrl NT and shKDM4C expressing U87 cells. α-Tubulin was used as an equal loading control. Protein expression levels were quantified against α-tubulin, and control levels were set at 1. The modification levels of p53 was quantified relative to p53.

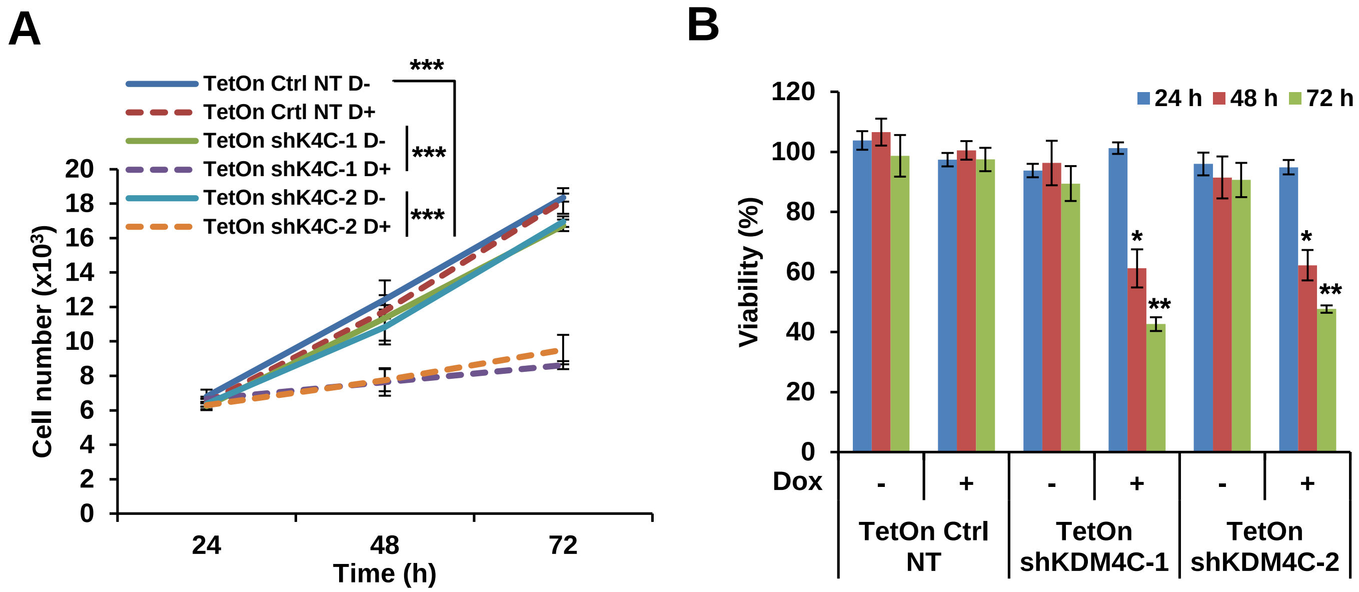

Fig. S15 Depletion of KDM4C inhibits glioblastoma cell growth and viability.
(A-B) Cell growth (A) and viability assay (B) of U87 TetOn-KDM4C cells expressing shCtrl NT or shKDM4C. Data represent mean ± SD (n = 3). *P < 0.05, **P < 0.01, and ***P < 0.001; one-way ANOVA analysis.
